# COVID Border Accountability Project, a hand-coded global database of border closures introduced during 2020

**DOI:** 10.1038/s41597-021-01031-5

**Published:** 2021-09-29

**Authors:** Mary A. Shiraef, Cora Hirst, Mark A. Weiss, Sarah Naseer, Nikolas Lazar, Elizabeth Beling, Erin Straight, Lukas Feddern, Noah Rusk Taylor, Cayleigh Jackson, William Yu, Aadya Bhaskaran, Layth Mattar, Matthew Amme, Maggie Shum, Mary Louise Mitsdarffer, Johanna Sweere, Susanna E. Brantley, Luis L. Schenoni, Colin Lewis-Beck, COBAP Team, Mary A. Shiraef, Jonathan Falcone, Sonila Hasaj, Amalia Gradie, Rachel E. Musetti, Thuy Nguyen, Yashwini Selvaraj, Bryn Walker, Matthew Amme, Cora Hirst, Sarah Naseer, Nikolas Lazar, Erin Straight, Lukas Feddern, Noah Rusk Taylor, Cayleigh Jackson, William Yu, Aadya Bhaskaran, Layth Mattar, Mark A. Weiss, Maggie Shum, Mary Louise Mitsdarffer

**Affiliations:** 1 Department of Political Science, University of Notre Dame, Notre Dame, Indiana USA; 2 Departments of Biology and Anthropology, Emory University, Atlanta, Georgia USA; 3 Departments of Political Science and Mathematics, Emory University, Atlanta, Georgia USA; 4 Department of Quantitative Theory and Methods, Emory University, Atlanta, Georgia USA; 5 Department of Economics, Brown University, Providence, Rhode Island USA; 6 Department of Sociology, Emory University, Atlanta, Georgia USA; 7 Center for Latin American, Caribbean, and Latinx Studies, Vanderbilt University, Nashville, Tennessee USA; 8 University Medical Center Hamburg-Eppendorf, Department of Health Economics and Health Services Research, Hamburg, Germany; 9 Department of Philosophy, Emory University, Atlanta, Georgia USA; 10 Nell Woodruff School of Nursing, Emory University, Atlanta, Georgia USA; 11 Department of Economics, Emory University, Atlanta, Georgia USA; 12 Departments of Biology and Middle Eastern and South Asian Studies, Emory University, Atlanta, Georgia USA; 13 COVID Border Accountability Project (COBAP), Austin, Texas USA; 14 University of Notre Dame, Keough School of Global Affairs, Notre Dame, Indiana USA; 15 Rutgers University, Department of Childhood Studies, Camden, New Jersey USA; 16 Charles River Associates, Boston, Massachusetts USA; 17 Department of Cell Biology, Duke University, Durham, North Carolina USA; 18 Universität Konstanz, Department of Politics and Public Administration, Baden, Württemberg Germany; 19 Department of Statistics & Actuarial Science, University of Iowa, Iowa City, Iowa USA; 20 Faculty of Public Affairs, Carleton University, Ottawa, Ontario Canada; 21 Department of Economics, Vanderbilt University, Nashville, TN USA; 22 Center for the Study of Human Health, Emory University, Atlanta, GA USA; 23 Department of Environmental Science, Emory University, Atlanta, GA USA; 24 Department of Political Science, University of Oregon, Eugene, Oregon USA; 25 Rikkyo University, Toshima City, Tokyo Japan; 26 Department of History, Emory University, Atlanta, GA USA

**Keywords:** Politics, Viral infection

## Abstract

Quantifying the timing and content of policy changes affecting international travel and immigration is key to ongoing research on the spread of SARS-CoV-2 and the socioeconomic impacts of border closures. The COVID Border Accountability Project (COBAP) provides a hand-coded dataset of >1000 policies systematized to reflect a complete timeline of country-level restrictions on movement across international borders during 2020. Trained research assistants used pre-set definitions to source, categorize and verify for each new border policy: start and end dates, whether the closure is “complete” or “partial”, which exceptions are made, which countries are banned, and which air/land/sea borders were closed. COBAP verified the database through internal and external audits from public health experts. For purposes of further verification and future data mining efforts of pandemic research, the full text of each policy was archived. The structure of the COBAP dataset is designed for use by social and biomedical scientists. For broad accessibility to policymakers and the public, our website depicts the data in an interactive, user-friendly, time-based map.

## Abstract (summary)

**Table:** 

Measurement(s)	border policy
Technology Type(s)	digital curation
Factor Type(s)	country • temporal interval
Sample Characteristic - Environment	Coronavirus pandemic
Sample Characteristic - Location	global

Machine-accessible metadata file describing the reported data: 10.6084/m9.figshare.16438932

## Background & Summary

**Fig. 1:**
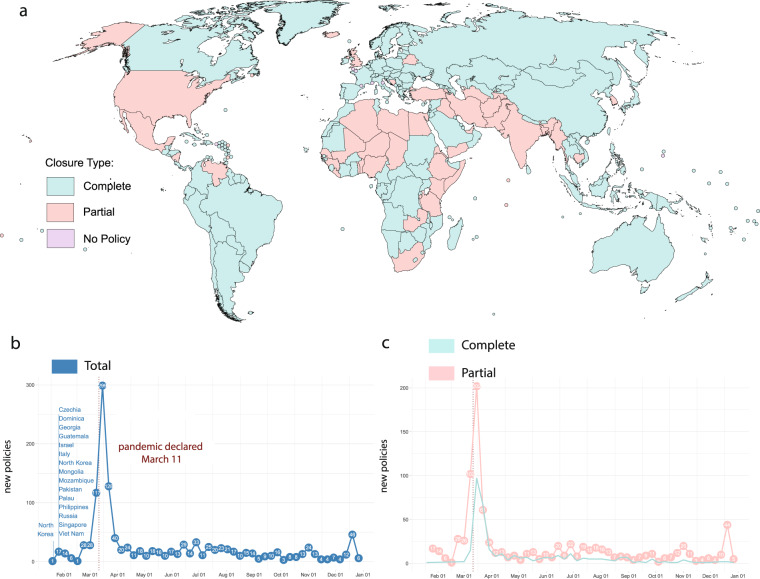
Scope of data collection. (**a**) Map of border closures enacted in total throughout 2020. The teal color indicates the countries that enacted complete closures at any point in 2020, and pink indicates the same for partial closures. If a country instituted both, we show the complete closure color. Countries for which there were no policies are purple. (**b**) New policy counts over time and an indication of the official start of the COVID-19 pandemic on 11-March 2020. The first countries to enact new policies are listed on the chart. (**c**) Breakdown of new complete (teal) and partial (pink) closures over time.

Since the first reported case of the novel coronavirus (SARS-CoV-2) crossing international borders (into Thailand on 13-Jan 2020)^1–3^, policymakers introduced sweeping public health measures (non-pharmaceutical interventions, or NPIs) at international borders—including restrictions on entry altogether. The first country-level restriction on movement across international borders was introduced on 22-Jan 2020 by North Korea. The number of border closures then increased between early February and early March, peaking at 299, just after the World Health Organization (WHO) declared the coronavirus a pandemic on 11-Mar (See Fig. 1b). As more data emerges on SARS-CoV-2 transmission and the health outcomes of those infected, national governments must weigh the costs of scaling back restrictions, introducing new ones, and/or lifting international travel restrictions.

The COVID Border Accountability Project (COBAP) fills a vital knowledge gap as the first database to document systematically the global time horizon of all new international border restrictions related to incoming human movement for the complete timeline of 2020. COBAP is one of two projects, to the authors’ knowledge, that focused exclusively on immigration-, travel-, and border-related policy changes. The most comparable project to ours is the Mobility and border control in response to the COVID-19 outbreak dataset^4^, which documented government decisions in European countries related to border closures and lockdowns from 1-Mar to 20-Jun 2020 (and are updating the database as resources allow). Another comparable resource is the COVID-19 Law Lab (https://covidlawlab.org/), which hosts policy texts related to pandemic management from >190 countries and has a category “Movement & Distancing Restrictions” with over 2000 entries. However, neither of these resources parse restrictions on entry from government recommendations to do so, whereas we focus on actual restrictions on entry. We aimed to cover all new closures introduced at international borders in response to COVID-19 for the complete timeline of 2020. We also differ in scope by covering the entire globe, including 104 island countries or territories. Furthermore, we provide our data in a user-friendly map visualization: (https://covidborderaccountability.org/). For further review of COBAP, in context with other datasets, see our Methods section below.

The most immediate use of the COBAP database is that it allows researchers to evaluate the effectiveness of various national-level policies aimed at slowing the spread of the virus with travel restrictions. Preliminary studies have examined the efficacy of government responses to ban travel, but do not implement the data breadth, quality, and precision of the COBAP dataset. Political scientists can use the data to evaluate the propensity of specific regime types—i.e., competitive authoritarian governments—to establish restrictions of a higher magnitude and impact during crises. Social scientists can use the COBAP dataset to evaluate if the capacity of states—i.e., their infrastructural power—renders certain policies more effective. As additional data becomes available, our country-level government response data serves as a baseline for addressing a wide range of pandemic research questions in both the social and biomedical sciences. Further uses are specified in the Usage Notes section.

## Methods

The COBAP team sourced, hand-coded, and archived >1000 country-level border policies introduced in response to the COVID-19 pandemic between January and December 2020. We implemented a ranked source approach with national-level governmental sources in the host country as our priority for each policy record. As proxies for a correct timeline of intricate travel and border restrictions that impacted multiple populations at a time, we incorporated in our database those made available to the public for a short period of time by the international airline industry and the international insurance industry. We added to these resources—with the use of RA interpretive labor and the Wayback Machine (https://archive.org/)—the date the policies were removed. The database incorporates a restricted portion of the “movement restrictions” recorded in the ACAPS COVID-19 Government Measures Dataset^5^, extracted systematically by an RA manager, and allotted into individual assignment spreadsheets for further interpretation by RAs [step-by-step restriction process described in the Supplementary Materials]. In limited cases, when policy texts were not available, but the RA was convinced of the policy implementation, we utilized social media announcements from verified government sources, or we combined sources from major news outlets of host countries and major news outlets from external countries.

### Review of comparable datasets

Before launching our data collection effort, we completed an extensive review of other data sources. Other existing datasets for tracking government responses to COVID-19 have trended toward a broader approach to data collection of travel restrictions^5–11^, a less systematic collection of travel restrictions^12–14^, a bias toward the needs of particular travelers^15–17^, or an incomplete time horizon of public information and scope of modes of movement^18–22^.

More specifically, the earliest resource which became available was the ACAPS COVID-19 Government Measures Dataset^5^, a crisis dataset for humanitarian workers which provided policies in five categories: social distancing, movement restrictions, public health measures, social and economic measures and lockdowns. However, the dates are not consistently recorded or verified, which is problematic for scientific analyses; and, similar to Piccoli *et al*.^4^, the category of “movement restrictions” is not parsed between actual border closures and travel recommendations. For instance, while reviewing systematically the ACAPS category of movement restrictions for inclusion into our dataset, we found substantive overlaps in the policies recorded in respective categories, i.e., national lockdowns; and moreover, we decided ACAPS primary reliance on media, external governmental, and non-governmental resources made the policy records too imprecise to use without extensive cleaning and further verification of dates. Any governmental source in the ACAPS dataset^5^ which met our pre-set criteria of an actual restriction on movement on entry into a country was merged into the COBAP dataset.

The next resources which became available were the CoronaNet Research Project^6^. Although more precise and expansive than ACAPS in their data categorization, CoronaNet’s approach resulted in a “living”, yet-to-be-verified database with similar source reliability issues to the ACAPS dataset.

The main differences between these two early resources and the COBAP dataset are that we have systematically verified, through internal auditing and source cross-checking, the dates in our data, completed comprehensive searches which prioritized host governments’ policy texts (instead of media or non-governmental sources), and parsed a more precise collection of enforced policies at international borders (and the countries targeted).

Another difference is that COBAP recorded and sourced end dates to policies, when applicable throughout 2020, whereas ACAPS did not, and we archived all our source links, whereas ACAPS and Coronanet^6^ did not. Coronanet is the only other project to the author’s knowledge that recorded some end dates. At the time of writing, Coronanet’s most comparable category to our dataset is called “external border restrictions” (restricting on the sub-categories of “total border crossing ban,” “visa restrictions” and “blanks”); this category contains recorded end dates for less than a third (306 out of 998) of their entries; but their start and end dates are difficult, and in some cases impossible, to verify because the source links are not archived or differentiated for start versus end dates. COBAP includes at least one archived source per end date (with 997 out of 1329 having end dates). We have not yet merged or cross-checked our dataset with Coronanet, but through outreach with Coronanet toward doing so, we recommend that users incorporate our verified start and end dates for Coronanet’s category “external border restrictions.”

Another difference, of particular interest to political scientists, is that the COBAP dataset records portions of the 2020 timeline for each country when citizens themselves were restricted from entry; no other project, to our knowledge, has done so. We also searched for and recorded policies that impacted immigrants specifically (through the cancellation of routine visa processing services).

Two non-academic resources that serve as a reliable proxy for international restrictions on movement are the International Air Transport Association (IATA)^16^, a for-profit organization that much of the airline industry relies on for real-time travel rules, and the International SOS (SOS)^17^, the largest international insurance company. However, on 27-May SOS put up a paywall to access this information; and on 6-Aug 2020, IATA did the same. For purposes of transparency in our sources, we incorporated information from IATA and SOS into our database only through the timelines in which the data was publicly available—which we archived via the Wayback Machine (https://archive.org/). Thereafter in 2020, we rely only on host governmental sources. We also cross-referenced the publicly available IATA and SOS information with government sources. Generally speaking, we found IATA to be a strong proxy source for air travel only; and SOS to be a helpful resource for land border closures. However, if the information conflicted against the government source, we record the dates and content of the government source.

Academic projects—such as HIT-COVID^7^, Complexity Science Hub COVID-19 Control Strategies List (CCCSL)^8^, and Response2covid19^9^,—emerged in 2020 to address the imprecise structures of the earliest data collection efforts. Most recently, the Oxford COVID-19 Government Response Tracker (OxCGRT)^10^ and the Worldwide Non-pharmaceutical Interventions Tracker for COVID-19 (WNTRAC)^11^ were released. The HIT-COVID dataset^7^ provides an excellent, comprehensive resource, reliable from (1) its internal auditing system for start dates and (2) its source collection transparency; however, for our topic of interest—international restrictions on movement—their dataset structure does not parse beyond border closures (air, land, or sea) and internal movement restrictions. In addition to air, land and sea closures, the COBAP dataset records the specific population groups whose incoming entry was restricted by each policy. We do not yet include internal movement restrictions. As such, for comparisons on policy efficacy with SARS-CoV2 data, we recommend combining our dataset for international border closures with HIT-COVID’s category of internal movement restrictions.

Another difference is that HIT-COVID’s^7^ sources rely extensively on the non-governmental source, GardaWorld (https://www.garda.com/), which we found to be unreliable on source transparency, especially for travel restrictions. COBAP initially incorporated many of GardaWorld’s listed policies in our dataset; but after finding inconsistencies in dates, compared to host governmental sources, we removed most GardaWorld sources and instead, rely primarily on governmental sources. Only in limited cases, we included a GardaWorld source with a second, independent source with matching information. Similarly, the CCCSL^8^ and Response2covid19^9^ datasets were structured to correct for the non-discreteness of the ACAPS^5^ categorization of NPIs; but rely mostly on sources different from host governmental texts, including GardaWorld. Response2covid19 relies almost entirely on ACAPS information and thus, contains the same sourcing issues.

The OxCGRT database^10^ archived their sources and effectively document end dates (by providing a helpful time-series data format with updates to policies and sources); however, their sources are primarily from GardaWorld and/or media-based sources. Furthermore, OxCGRT is a “live” database of tremendous importance for future pandemic research, but has portions of their 2020 timeline—i.e., their “legacy data”—not yet verified (https://github.com/OxCGRT/covid-policy-tracker/tree/master/legacy_data_20200425).

As such, for date precision, we recommend users to incorporate our dataset into the OxCGRT^10^ category “international travel controls” (restricting on 4 for what we call “complete closures” and/or on 3 for our “partial closures”). The difference to note here is that the category from OxCGRT does not distinguish between outgoing and incoming international movement or between screening measures and quarantines versus closures. COBAP’s database records restrictions strictly on incoming travel.

WNTRAC^11^ is our preferred approach of the above reviewed projects because combining AI-assisted text extraction with manual verification is more time efficient than manual verification of policies across the globe. This approach will allow a broader systematized collection of NPIs per country. However, WNTRAC relies on Wikipedia articles for their policy records, whereas for our topic of international travel restrictions, we found Wikipedia articles to be inadequate on its own. We also mined Wikipedia’s article on travel restrictions^13^ as well as the individual country-level pandemic response sections (although we did so manually). Roughly half of the sources were media-based rather than governmental. Most were archived. As we did with ACAPS, we included the policy if it met our pre-set criteria for an actual movement restriction on movement across international borders. Different from WNTRAC, COBAP verified the dates and policy content from the actual governmental texts and/or industry data, as well as outreach to public officials when information conflicted. WNTRAC also does not have end dates in their currently available dataset, and if Wikipedia is their primary source for this (by time of writing), we have not yet found Wikipedia to have entered content for end dates or “re-openings.” The difference in the COBAP dataset is that we prioritize end dates, and source them with domestic media searches, as necessary.

Overall, the main differences between these pre-existing projects and COBAP are (1) the scope of countries covered, (2) source prioritization, and (3) precision in recording actual restrictions on entry (and which population groups were restricted).

### Scope comparison to other projects

Regarding scope, for international border closures, HIT-COVID^6^ recorded 412 policies introduced by 83 countries for the timeline of Jan-Jul 2020; during the same period, we recorded 1062 policies from 235 countries (or administrative units of island territories). Regarding source quality, HIT-COVID’s policy records rely on a wide array of non-governmental sources (and do not distinguish source type), whereas COBAP prioritized the policy texts produced by the responsible entity in the host country. COBAP ranks data quality by source-type. For international border closures, CCCSL^7^ provide a category of “travel restrictions” (restricting on the subcategories of border restrictions, airport restrictions and port and ship restrictions), which covers a longer timeline than HIT-COVID (Jan-Dec 2020), but with far fewer country-level policies (479) and for far fewer countries (66). Coronanet’s^6^ policy timeline, like ours, spans the 2020 timeline; they record for international border closures ~1000 policies for 182 countries. OxCGRT’s^10^ comparable category to ours—“international travel controls” (restricting on 4 and/or on 3)—includes ~3000 updates for 180 countries. We did not mine these because they were not consistently differentiated between actual restrictions on entry (versus other measures) or on directionality (between outgoing or incoming traffic). WNTRAC’s^11^ comparable category—international flight restrictions—includes 474 policies for 124 territories. After prioritizing extensive searches to include available data on all inhabited island territories and for countries with shorter histories of providing consistent public information, the COBAP data collectors recorded (by the time of writing) 1312 policies for 246 countries or territories within the 2020 timeline.

### Policy content comparison to other projects

Regarding the precision of the content of the COBAP database, the topic of international border closures was not the focus of most NPI data collection efforts. Our dataset focuses on international border closures. This focus allowed us to prioritize our source quality and verification process, whereas projects which took a broad approach to collect all NPIs need additional verification. For instance, WNTRAC^11^ manually checked only the text as recorded in Wikipedia, whereas we verified dates from each available governmental text. Through manual text interpretation and verification, we provide a consistently parsed distinction in complete versus partial closures, the names of countries impacted by each policy, and further distinctions under partial closures, including whether incoming travel was restricted through air/land/sea, through a travel history ban versus a citizenship ban, or through new visa restrictions. To the author’s knowledge, at the time of writing, the COBAP dataset provides the most comprehensive *and* the most precisely parsed collection of international border closures introduced in response to COVID-19.

In sum, the novel contributions of our dataset are that (1) we record, in systematic detail, a comprehensive set of the different types of movement restrictions implemented, a correct start and end date, and specifically which populations were targeted by (or excepted by) each new policy between Jan to Dec 2020. (2) We depict our updated data and sources in an interactive map visualization (https://covidborderaccountability.org/), combined with Dong *et al*.’s (2020) COVID-19 case and mortality data over time^23^. (3) To the author’s knowledge, COBAP is the first research team to record systematically the governmental responses to COVID-19 related to immigration at the global level. (4) We also record policy information on 246 countries and associated island territories, pulling ahead of the number of countries covered by other open data projects in the friendly competition of this collection in *Scientific Data*. (For the full country list we cover, see Supplementary Materials). (5) We will continue data collection and review through Nov 15 2021, and as resources allow.

### Data collection process

**Fig. 2:**
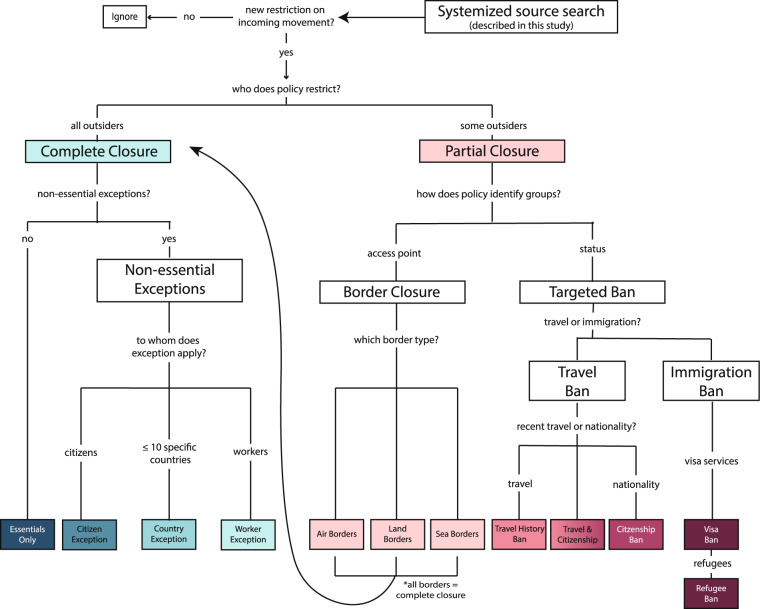
Overview of data collection process. The flow chart presents the process by which RAs identified, classified, and coded each policy. Complete definitions for each sub-category can be found in Online-only Table 1.

We designed our data collection process with a standardized decision set (Fig. 2) built into a carefully designed Qualtrics survey (Qualtrics.com) which prodded RAs to prioritize source quality and rely on pre-set definitions. These definitions are viewable in Online-only Table 1. To maximize the utility of the dataset for both social and biomedical scientists, the gradation of the data collection includes important information for designing an experiment, such as listed exceptions to complete closures and names of the specific populations targeted by partial restrictions. Our internal data verification process prioritizes valid start and end dates, a crucial feature of data for examining whether specified durations of the policies correspond with drops in case and mortality data for SARS-CoV-2. Our sources for each interpreted policy text are recorded on the Wayback Machine (https://archive.org/) or Permalink (https://perma.cc/) for lasting transparency of the data collection process, and for preserving capacity for further data scraping.

Our data collection process begins with a search at the country-level for any new policy restrictions that relate to international border travel or immigration. We organize these policies into two overarching categories of cross-border restrictions: “complete closures” and “partial closures.” A complete closure is a new policy in which all newcomers are banned from all ports of entry—AIR, LAND, and SEA—with limited exceptions, including citizens, nationals from a specified country or set of up to 10 countries, and/or essential reasons, e.g., health emergencies, extreme humanitarian/diplomatic reasons, dignitaries, cargo flights, commercial transport, essential deliveries, permanent residents, existing visa holders, and family members of citizens. A partial closure is a new policy that restricts access of specific groups of people, whether by certain nationalities, travel histories; those entering through a specified land, sea, or air border; OR all land borders closed OR all air borders closed OR all sea borders closed (but not all three). See the final count of complete versus partial closures in Fig. 1.c. An overview of the data organization process into subcategories is provided below in Fig. 2. Our subcategory definitions are provided in Online-only Table 1.

To distinguish systematically between travel and immigration-related restrictions, the PI curated a 21-question Qualtrics survey with a built-in decision tree (see overview in Fig. 2). She recruited volunteer data collectors primarily from her professional network of academic institutions. For the first phase of data collection (covering January—August 2020), a team of eighteen trained data collectors piloted a Qualtrics survey for recording all international border policies. To ensure a comparable approach to data collection across the globe, we assigned data collectors to record the policies of at least five specific countries. To avoid bias in the data, the PI assigned countries both in and outside of their knowledge bases and language expertise. For countries in which the RA was assigned a country for which s/he did not read the country’s official language(s), s/he was assigned a policy expert and/or language consultant who answered questions about policies which the RA could not resolve with the use of Google Translate (translate.google.com).

The policy definitions implemented in the piloted Qualtrics survey of the first phase remained unchanged throughout data collection [Supplementary Materials]. The first part of the survey is designed to guide RAs to locate the best possible source for the policy introduced. The second portion of the survey helps the RA to restrict our search to national-level restrictions which impact migrants or commercial travel and decide systematically between two umbrella categories: complete versus partial closures, followed by further sub-category refinement with information we expect to be broadly useful for both the biomedical and social sciences.

When an international border policy restricts all newcomers, we recorded “complete closures” without any exceptions (essentials_excep), “complete closures” with citizen exceptions (citizen_excep), complete closures with specific country(ies) exceptions (country_excep), and complete closures with workers exceptions (workers_excep); when the policy restricts on a subset of newcomers, we record partial closures which relate to access points (border_closure) or partial closures which relate to status (targeted_ban). We break targeted bans further into travel bans and immigration bans. Travel bans are restrictions based on recent travel history (travelhistory_ban) or nationality (targeted_ban); immigrations bans, which impact those seeking regular visa services (visa_ban), targeted at all or specific foreign population groups; or they restrict refugees or asylum seekers (refugee_ban).

We do not record measures such as: travel advisories, unofficial immigration restrictions, repatriation flights, individual incidents, or restrictions on cruise ships. Instead, we focused on actual border closures, which allowed us to collect more precise data on more countries and their associated territories than pre-existing projects. We also have higher confidence in the data, having manually reviewed and confirmed the information recorded for each policy at least twice by separate individuals. After that, we reviewed the database categorically with one RA assigned per category. At the time of writing, we are confident we have reviewed all available resources, to our knowledge with the goal of depicting all new restrictions on incoming human movement, across an international border, introduced at the country-level (and its duration) for the complete timeline of 2020. In practice, this interpretation process was difficult; for confusing policy texts, we worked in pairs, raising, and resolving issues in real-time on an open Slack channel (slack.com) [See “Frequently Asked Questions” (FAQ) in Supplementary Materials]. The process resolution most often was managed through the use of our pre-set definitions.

For the second phase of data collection (covering September—December 2020), we prioritized governmental sources and recruited and trained ten additional RAs. Each of the RAs completed a full contextual search on their assigned countries, including (1) the official name and website for the governing body(ies) responsible for national-level policies which relate to travel and/or immigration; (2) the official national-level coronavirus website per country; (3) whether the country is an island and; (4) whether a land border exists for the island country. The country-level COVID-19 response websites and governing body websites are reflected in the pop-up function of our country-level website visualization (https://covidborderaccountability.org/). This contextual search per RA served as a project resource which RAs were responsible to check for new policy changes each week and to decide if the change met the standards of inclusion for the COBAP database.

The widespread differences amongst country-level infrastructures for governing travel, as well as the variation in clear and consistent messaging per country, necessitated pre-set criteria for consistency in the database.

### Standards for inclusion

COBAP restricts the database to include policies which were implemented by the government or administrative structure of the country or territory. If the country produced a policy text which related to stopping travel or immigration across international borders, we aimed to include it (as either a complete or partial closure). We limit the scope of our data collection in this way to provide a framework that amplifies the nation-level decision against the granularity of the population groups impacted per policy. Although time-consuming, this approach was beneficial in that it avoided the duplication of data, recording incomparable data, as well as mis-recording of policies at the national level.

A potential vulnerability of our dataset is that it does not include cases in which a country may effectively have experienced a border closure (or drastic human movement reduction) due to another country’s policy decisions, or due to internal national-level or subnational level lockdowns. Furthermore, we do not capture the full extent of policies which are multidirectional, i.e., when one nation closes a border to both outgoing and incoming traffic. We also do not capture quarantine requirements for persons entering a country, restrictions on cruise travel, or rules regarding passenger transit. For instance, when Australia’s government bars entry to all foreigners, but allows specific nations’ citizens to transit through Australia via commercial air travel, we record this as a “complete closure” (no countries exceptions) since these populations are not able to enter and stay. As such, for the full scope of reduction on all human movement in a given location, we recommend combining our database with de-identified mobile phone data^23^. For comparing outcomes of international border restrictions with other measures, we recommend combining the COBAP database with the HIT-COVID NPIs, given their comprehensive scope and high source quality. The description of each dataset column is viewable in Online-only Table 2.

### Confusing Contexts

Policy interpretation based on decisions made by national-level governing entities is, in practice, quite complicated. Despite variation in state capacity, our goal was to approach national-level governing territories systematically to produce correct data. Given the variation in administrative structures for travel governance, the contexts were often complicated, and decisions had to be documented. Examples of the complicated cases we faced, and the decisions we made to synthesize the data, include:

Prior to 2020, the Schengen Area in Europe operated as an agreement amongst 26 countries to guarantee freedom of movement. However, individual countries can choose to introduce internal border controls if warranted. As such, we decided to collect both the Schengen Area recommendations on travel and individual countries’ restrictions. The Schengen Area recommendations are the only policies in our database which are not actual restrictions on entry.

French national-level policies apply to French overseas departments and regions (which are territorially not connected to mainland France). We do not include these national-level policies, for these overseas French entities but record any different policies they introduced separately from mainland France. (We faced a similar problem with the U.S. in relation to Guam and Puerto Rico; and took the same approach.)

Guernsey’s state-owned airline, Aurigny, suspended all flights, which effectively removed all commercial air access to Guernsey (except emergency service to Southampton). We did not record this as air border closure because Guernsey’s government never explicitly banned arrivals.

Vatican City was closed to all foreigners, but workers who live in Rome are allowed to travel into the Vatican City for work purposes. We record this as a complete_closure, with a workers_exception.

Niuean, Cook Islander, and Tokelauan citizens travel on New Zealand passports as New Zealand citizens. This means when we record a “complete closure” for New Zealand, we might not capture a specific country exception for these nations. We note this as a potential omission, as our general approach was to only record the country names listed in the text (for exceptions and bans).

For additional complicated cases, and the decisions we made to address them, see our RA FAQ list (Supplementary Materials). As a general, pre-set distinction, we allowed in our definition for “complete closures” an exception for up to 10 countries, and we recorded as “partial closures” any policy that allowed the population groups from more than 10 countries to enter on a given date. We chose the number 10 for consistency in the data; but researchers can adapt this easily for their purposes because we also record the names of each country barred from entry (for partial closures) or for those which were provided an exception (for complete closures).

To represent the data in a visually accessible way, COBAP also introduced a web map tool to view policies over time. This tool uses the Carto library and JavaScript to represent the changes in policy over time on a choropleth map. The data visualizations in Fig. 1 were built using R, categorizing policies by type over time. All code is available on our project GitHub (https://github.com/COBAPteam/COBAP).

## Data Records

The raw outputs of the Qualtrics surveys are processed each week by removing policies that have been corrected or updated with new information. The standardized ISO 3/ISO 2 country code is attached to each policy to simplify country identification. This step renders a complete list of policies recorded with a unique ID per policy.

The sanitized output of the Qualtrics surveys is combined via a Python script to automate output of survey responses in a more machine-readable data framework listed below. The script also appends new policy end dates, whose sources differ from the start dates. This verified version of the COBAP dataset is hosted on *Harvard Dataverse*^24^. This process also assists with the (1) verification process for validating new end dates and web links and (2) production of other data outputs used for the interactive, time-based visualization available on our project website (https://covidborderaccountability.org/).

## Technical Validation

Recording an unprecedented phenomenon of global policy adaptations to a novel infectious disease, with a new international team, presented unique challenges to the data collection and verification processes. COBAP is grateful to other comparable projects that took an open data and open process approach^4–16,20–22,25^, which helped to identify early strategies to work effectively across multiple time zones and collect data in multiple languages. Different from our larger partner projects, we chose to have a small team cover more countries (than to assign one RA per country).

We found it improved the data quality to assign at least five countries to each trained RA for data collection, and at least five different countries to the same RA for data verification. The use of a central Slack channel (slack.com) for addressing real-time questions and the implementation of forced question responses via Qualtrics surveys (qualtrics.com) also contributed to high initial data quality. Finally, accountability in the data quality is high with authorship attached for RAs who cover five or more specific countries over an agreed-upon period of time (see “contributors expectations” in Supplementary Materials).

Coding decisions were verified by a second RA by clicking on the source link and ensuring the information is correct. Disputes were discussed between the RAs and resolved and reviewed by the PI.

**Fig. 3:**
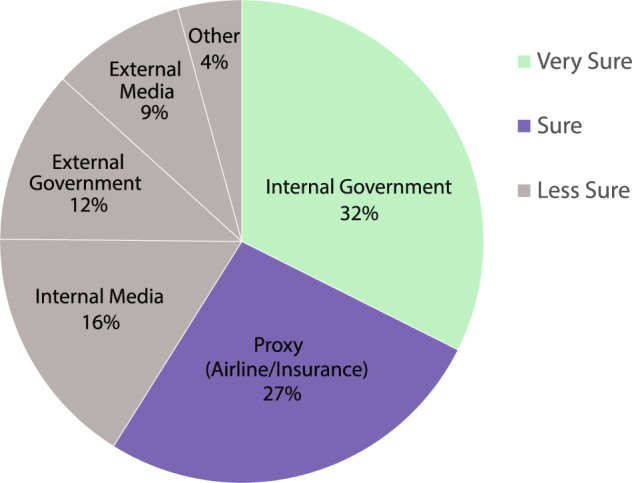
Data Quality Breakdown by source type. Chart denotes the reliability of data per country in the COBAP dataset (through Dec 2020). Countries whose data use only governmental sources, we rank as “very sure” (blue). For those we could not find governmental sources but found reliable proxies, we rank in the dataset as “sure” (orange). Countries where we could not find governmental or proxy sources for, we rank as “less sure” with a breakdown of the sources used (grey).

The data review process includes:

1. **end date verification**: a check on all policies recorded by another RA that do not yet have end dates. Combining a search of the initial policy text, the project resources on the country and Google’s search engine (Google.com), the reviewer adds new end dates herself in a second Qualtrics survey (Qualtrics.com) and justifies the decision with the source. For end dates, we only use government sources, or in some cases, a set of reputable media sources indicating borders were opened.
2. **policy content verification**: a review of past coding decisions for each policy a different RA recorded, recording errors and sending to initial RA to fix.
3. **database updates**: a manual fix of any issues found by a reviewing RA.
4. **PI review**: a review of unresolved issues between two RAs via a PI-moderated open Slack channel.
5. **crowdsourcing and country expert outreach**: The PI reached out with the help of personalized emails, a news publication, and Twitter (https://twitter.com/COBAPteam) to country experts for countries whose data she and the responsible RAs were unable to verify. RAs also utilized their professional networks, reached out to the public entity directly with clarifying questions, and resolved issues or omissions in the database accordingly.
6. **data quality ranking per country**: for countries whose data we have used only governmental sources we found to be reliable, we rank as “very sure.” For those we could not find governmental sources but found reliable proxies, we rank in the dataset as “sure.” For countries we could not find governmental or proxy sources for, we rank as “less sure.” See Fig. 3 for source type breakdown.

## Usage Notes

On its own, the COBAP dataset is the most useful for research questions related to policy efficacy of national-level decisions made during a crisis. For instance, our team has already combined the COBAP data with available COVID-19 case data to investigate whether restrictions on travel were effective in reducing the viral spread between the restricted populations. For an experimental design, we recommend combining the COBAP data with other country-level datasets to control for other types of measures introduced at the same time, as well as underlying differences between countries, such as healthcare capacities. The granularity of the database, which parses specific country/population exceptions, allows a researcher to discern the impacts of different levels of movement restrictions on the viral spread. These distinctions—especially amongst air, land and sea travel—may offer insight on viral “entry-points” into a given region.

Epidemiological analyses are facilitated by the COBAP website inclusion of the number of COVID cases and deaths for each country over time^26^. We do not recommend, however, drawing causal claims from this early correlational data. Instead, we suggest using our visualized correlational data alongside other indicators per country to implement an experimental design with additional data becoming available.

Of interest to virologists from the COBAP dataset are the impacts of travel restrictions on the spread of novel viral strains, where the virus’ genetic code has changed due to mutations. An ongoing concern in the COVID-19 pandemic is the rise of a more virulent strain that current vaccines or diagnostic tests will not recognize, and that this strain will spread more rapidly. Tracking the spread of specific strains to a ‘host’ or ‘origin’ location is an arduous task, facilitated by a database that records restrictions or barriers to inter-population travel. Specifically, the COBAP database of border closures helps narrow the potential “paths” that a novel viral strain could have taken to arrive in a particular country.

For policymakers, our acute attention to start and end dates for policies throughout the 2020 timeline allows the question: at what point, during a pandemic, should border closures be considered (if at all), compared to expanded requirements for testing and/or quarantine? Further, the code for our country-level time-based data visualization could be adapted to visualize other datasets with more granular data on governmental responses at the state, county, or city-levels.

In sum, the COBAP database will assist both biomedical scientists with modeling viral transmission patterns for future pandemics and social scientists and policymakers with assessment on whether prior travel restrictions were effective. Given the versatility and wide-ranging implications of the COBAP dataset, the authors commit to fixing any errors submitted about policies introduced between Jan 2020 and Dec 2020 as soon as possible. At time of writing, the 2021 data is collected and up-to-date through Jun 15 2021–but not yet verified. We plan to record end dates to all policies introduced in 2020; and as resources allow, we will collect and verify new policies introduced in 2021. Please submit any issues on the data via our project GitHub.

## Supplementary information

Supplementary Information

## Data Availability

Codes for the database, raw data outputs and data visualizations appearing on our website are available on our project GitHub (https://github.com/COBAPteam/COBAP). The full text of the survey used to code each policy is available in the Supplementary Materials.

## Online-only Tables

**Online-only Table 1:** Data dictionary of International Movement Restrictions introduced in response to COVID-19.

Policy Type	Description	Policy Sub-type	Description	Policy Amount (to date) (n = 1,305)
**1. Complete Closure**	**a new policy in which all newcomers are banned with limited exceptions**	**1.1 Essentials-only Exception**	**a complete closure with exceptions for essentials but not for citizens**	**n = 38**
		**1.2**	**a complete closure with exceptions for essentials and for citizens (including citizens, permanent residents, and/or the family members of citizens and permanent residents)**	**n = 182**
		**Citizenship exception**		
		**1.3**	**a complete closure with exceptions for essentials and for nationals from a specific country or listed set of countries (up to 10)**	**n = 61**
		**Country(ies) exception**		
		**1.4**	**A complete closure with exceptions for specific work permit status holders**	**n = 89**
		**Workers exception**		
**2**	**a new policy which restricts access of specific groups of people**	**2.1**	**A partial closure which bans the application for new visas, whether all visa seekers or impacting those from specified countries**	**n = 63**
**Partial Closure**		**Visa Ban**		
		**2.2**	**A partial closure which bans foreign nationals from one country or group of countries, e.g. “entry to the country is denied to foreign nationals from Austria, Belgium, and France”**	**n = 99**
		**Citizenship Ban**		
		**2.2 and 2.3**	**Both 2.2 and 2.3**	**n = 15**
		**2.3**	**A partial closure which bans travelers who, regardless of nationality, have recently travelled through or from a specified country or group of countries, e.g. for “All travelers who have been to or travelled through China, Hong Kong, Iran, Italy, and Japan are advised to not enter the country, and may be denied entry”**	**n = 150**
		**Travel History Ban**		
		**2.4 Refugee Ban**	**A partial closure which uses language targeting “refugees” or “asylum seekers”**.	**n = 3**
		**2.5**	**A partial closure which impacts those entering through a specified land, sea or air border; OR all land borders closed OR all air borders closed OR all sea borders closed (but not all three)**	**n = 612**
		**Border Closure**		
**3. No Policy Implemented**	**A thorough search reveals no national level policies implemented that meet the criteria for a complete or partial closure**.			**n = 8**

**Online-only Table 2:** Description of dataset columns.

**id**	unique ID used for each policy
**country_name**	country name that implemented the restriction
**iso3**	unique three-letter country code published by the International Organization for Standardization. Non-standard codes: XKX - Kosovo, SOL - Somaliland, EUR - European Union Schengen Zone
**iso2**	unique two-letter country code published by the International Organization for Standardization. Non-standard codes: XK - Kosovo, XS - Somaliland, EU - European Union Schengen Zone
**policy_type**	one of COMPLETE, PARTIAL, or NOPOLICYIMPLEMENTED
**policy_subtype**	The policy sub-type, one of: ESSENTIAL_ONLY, CITIZEN_EXCEP, SPECIFIC_COUNTRY_EXCEP, WORK_EXCEP, VISA_BAN, CITIZENSHIP_BAN, HISTORY_BAN, REFUGEE_BAN, BORDER_CLOSURE, NONE. Note: Only the most restrictive partial closure is included, so a policy with CITIZENSHIP exception may have travel history or specific border closure information in the related fields.
**start_date**	the date the policy was implemented (DD_MM_YY)
**end_date**	the date the policy was lifted (DD_MM_YY)
**air**	whether there was an air border closure (1) or not (0)
**air_type**	whether the partial closure closed all or some air routes. One of: All, Specific, NA
**targets_air**	which air routes were targeted
**land**	whether there was a land border closure(1) or not (0)
**land_type**	whether the partial closure closed all or some land routes. One of: All, Specific, NA
**targets_land**	which land routes were targeted
**sea**	whether there was a sea border closure(1) or not (0)
**sea_type**	whether the partial closure closed all or some sea routes. One of: All, Specific, NA
**targets_sea**	which sea routes were targeted
**citizen**	whether the partial closure targets certain groups of travelers by citizenship (1) or not (0)
**citizen_list**	which groups were targeted based on their citizenship status
**history**	whether the partial closure targets certain groups of travelers by travel history (1) or not (0)
**history_list**	which groups were targeted based on their recent travel status
**refugee**	whether the partial closure uses the language of “refugee” or “asylum seeker” (1) or not (0)
**refugee_list**	which refugees are targeted
**visa**	whether visa seekers are targeted (1) or not (0)
**visa_type**	which visa seekers are targeted. One of: All, Specific, NA
**visa_list**	which visa seekers are targeted
**citizen_excep**	whether the complete closure makes an exception for citizens (1) or not (0)
**citizen_excep_list**	which persons are exempted from the complete closure
**country_excep**	whether specific country(ies) are exempted from the complete closure (1) or not (0)
**country_excep_list**	which country(ies) are exempted from the complete closure
**work_excep**	whether the complete closure exempts workers (1) or not (0)
**source(0-4)**	Web link to source of policy, one per column
