# Supplementary material for: COVID Border Accountability Project, a hand-coded global database of border closures introduced during 2020
Source: Sci Data. 2021 Sep 29;8:253. doi: 10.1038/s41597-021-01031-5 (PMC8481472; doi:10.1038/s41597-021-01031-5)
Supplement: Supplementary file 1 — Supplementary Information [file 41597_2021_1031_MOESM1_ESM.pdf]

COVID Border Accountability Project (COBAP)

Supplementary Materials: Research Assistant (RA) Manual

covidborderaccountability.org

For project updates, subscribe here. Questions? Contact nikolas_lazar@brown.edu.

Contents

1 Project Overview **2**

2 Conceptual Framework: Pre-set Definitions **2**

3 RA Assignments and Training **3**
3.1 COBAP Country List 3
3.2 Schengen Area 5
3.3 COBAP Country Code List 5
3.4 RA Policy Search 5
3.5 Policy Assignments from the RA Manager 6
3.6 Assignment Sheets for RAs 7

4 Qualtrics Surveys **8**
4.1 Qualtrics survey 1: adding new policies 8
4.2 Qualtrics Survey 2: adding end dates 10
4.3 Using Both Surveys 1 and 2 to Record Policy Changes 11

5 RAs Frequently Asked Questions (FAQs) **11**

6 Contributor Expectations **14**

7 The Names behind the Data **15**

8 Appendix **17**
8.1 Full Text of Qualtrics Survey 1 17
8.2 Full Text of Qualtrics Survey 2 32

1 Project Overview

From the emergence of a novel coronavirus disease in late 2019 to its prolific spread across the globe throughout 2020, the COVID-19 pandemic spurred widespread policy changes at the national and sub-national levels. In response, the COBAP Team curated a systematized dataset of new country-level restrictions on movement across international borders, introduced referencing COVID-19.

The weekly data collection process was carried out by trained research assistants (RAs) responsible for finding and recording all the policies introduced by at least five countries during the 2020 year. RAs were aided in their process with (1) a list of evolving project resources to review each week for new policies; (2) an RA Manager, who identifies and assigns potential policies to be reviewed for inclusion into the COBAP dataset; and (3) a curated Qualtrics survey, with which RAs locate the ideal source text, archive it on the Wayback Machine, read the text, and categorize the policy type according to pre-set policy definitions.

2 Conceptual Framework: Pre-set Definitions

The COBAP database systematized country-level restrictions on entry across international borders. A decision tree guided each step of the data coding process. First, an RA asked, with a policy text in hand: is this a national-level policy which limits movement of humans across international borders? If so, s/he included the policy in our database. Next, the RA asked: is this policy a complete or partial closure, with the following definitions in front of her.

Complete Closure: a new policy in which all newcomers are banned from all ports of entry—AIR, LAND, and SEA—with limited exceptions, including citizens, nationals from a specified country or set of up to 10 countries, and/or essential reasons, e.g. health emergencies, extreme humanitarian/diplomatic reasons, dignitaries, cargo flights, commercial transport, essential deliveries, permanent residents, existing visa holders, and family members of citizens.

If the RA decided the policy is a complete closure, s/he next decided the following sub-categories related to exceptions:

- Does the policy make an exception for citizens (including citizens, permanent residents, and/or the family members of citizens and permanent residents)? → **Citizen Exception**
- Does the policy make an exception for nationals from a specific country or listed set of countries (up to 10)? → **Country(ies) Exception**
- Does the policy make an exception for non-citizen work permit status holders? → **Workers Exception**
- If the RA indicates none of these exceptions in the survey record, COBAP automatically records → **Essentials-only Exception**

Partial Closure: a new policy which restricts access of specific groups of people, whether by certain nationalities, travel histories; those entering through a specified land, sea or air border; OR all land borders closed OR all air borders closed OR all sea borders closed (but not all three).

If the RA decided the policy is a partial closure, s/he next decided the following sub-categories related to the specific country population impacted by the ban:

- Does the policy ban foreign nationals from a specified country (or group of countries)? (i.e."entry to the country is denied to foreign nationals from Austria, Belgium, and France") → **Citizenship-based Ban**
- Does the policy ban travelers who, regardless of nationality, have recently travelled through or from a specified country or group of countries, (i.e. "All travelers who have been to or travelled through China, Hong Kong, Iran, Italy, and Japan are denied entry". → **Travel-based Ban**
- Does the policy restrict travelers who are seeking access to new visas, whether all visa seekers or impacting those from specified countries. → **Visa-based Ban**
- Does the policy restrict persons using the language "refugee" or "asylum seekers." → **Refugee-based Ban**
- Does the policy restrict travel through a specified land, sea or air border; OR all land borders closed OR all air borders closed OR all sea borders closed (but not all three) → **Border Closure**

3 RA Assignments and Training

3.1 COBAP Country List

Each RA was assigned a set of at least five countries selected from the following list (n=244).

Afghanistan, **Åland Islands**, Albania, Algeria, American Samoa, **Andorra**, Angola, Anguilla, Antarctica, Antigua and Barbuda, Argentina, Armenia, Aruba, Australia, **Austria**, Azerbaijan, Bahamas, Bahrain, Bangladesh, Barbados, Belarus, **Belgium**, Belize, Benin, Bermuda, Bhutan, Bolivia, Bonaire Sint Eustatius and Saba, Bosnia and Herzegovina, Botswana, Brazil, British Indian Ocean Territory, Brunei Darussalam, Bulgaria, Burkina Faso, Burundi, Cape Verde, Cambodia, Cameroon, Canada, Cayman Islands, Central African Republic, Chad, Chile, China, Christmas Island, Cocos (Keeling) Islands, Colombia, Comoros, Congo, Democratic Republic of the Congo, Cook Islands, Costa Rica, Côte d'Ivoire, Croatia, Cuba, Curaçao, Cyprus, **Czech Republic**, **Denmark**, Djibouti, Dominica, Dominican Republic, Ecuador, Egypt, El Salvador, Equatorial Guinea, Eritrea, **Estonia**, Eswatini, Ethiopia, Falkland Islands (Islas Malvinas), Faroe Islands, Fiji, **Finland**, **France**, French Guiana, French Polynesia, Gabon, Gambia, Georgia, **Germany**, Ghana, Gibraltar, **Greece**, Greenland, Grenada, Guadeloupe, Guam, Guatemala, Guernsey, Guinea, Guinea-Bissau, Guyana, Haiti, **Holy See (Vatican City State)**, Honduras, Hong Kong, **Hungary**, **Iceland**, India, Indonesia, Iran, Iraq, Ireland, Isle of Man, Israel, **Italy**, Jamaica, Japan, Jersey, Jordan, Kazakhstan, Kenya, Kiribati, North Korea, South Korea, Kuwait, Kosovo, Kyrgyzstan, Lao People's Democratic Republic, **Latvia**, Lebanon, Lesotho, Liberia, Libya, **Liechtenstein**, **Lithuania**, **Luxembourg**, Macao, North Macedonia, Madagascar, Malawi, Malaysia, Maldives, Mali, **Malta**, Marshall Islands, Martinique, Mauritania, Mauritius, Mayotte, Mexico, Micronesia Federated States of, Republic of Moldova, **Monaco**, Mongolia, Montenegro, Montserrat, Morocco, Mozambique, Myanmar, Namibia, Nauru, Nepal, **Netherlands**, New Caledonia, New Zealand, Nicaragua, Niger, Nigeria, Niue, Norfolk Island, Northern Mariana Islands, **Norway**, Oman, Pakistan, Palau, Palestine State of, Panama, Papua New Guinea, Paraguay, Peru, Philippines, Pitcairn, **Poland**, **Portugal**, Puerto Rico, Qatar, Réunion, Romania, Russian Federation, Rwanda, Saint Barthélemy, Saint Helena Ascension and Tristan da Cunha, Saint Kitts and Nevis, Saint Lucia, Saint Martin (French part), Saint Pierre and Miquelon, Saint Vincent and the Grenadines, Samoa, **San Marino**, Sao Tome and Principe, Saudi Arabia, Senegal, Serbia, Seychelles, Sierra Leone, Singapore, Sint Maarten (Dutch part), **Slovakia**, **Slovenia**, Solomon Islands, Somalia, Somaliland, South Africa, South Sudan, **Spain**, Sri Lanka, Sudan, Suriname, Svalbard and Jan Mayen, **Sweden**, **Switzerland**, Syrian Arab Republic, Taiwan, Tajikistan, United Republic of Tanzania, Thailand, Timor-Leste (East Timor), Togo, Tokelau, Tonga, Trinidad and Tobago, Tunisia, Turkey, Turkmenistan, Turks and Caicos Islands, Tuvalu, Uganda, Ukraine, United Arab Emirates, United Kingdom, United States of America, Uruguay, Uzbekistan, Vanuatu, Venezuela, Viet nam, Virgin Islands British, Virgin Islands U.S., Wallis and Futuna, Sahrawi Arab Democratic Republic (Western Sahara), Yemen, Zambia, and Zimbabwe.

3.2 Schengen Area

In the list above, countries which agree to follow **Schengen Area** rules (n=26) are marked in bold, as are those with open borders with the Schengen Area (Andorra[1], Monaco, San Marino, and Vatican City)[2]. Schengen recommendations are the only "recommendations" included in the database; and are recorded as separate policies from those introduced by member-states. For policies that restrict entry from all countries but this list, we use the tag NONEU in the targeted field.

3.3 COBAP Country Code List

We follow the International Standard for Organization (ISO) 3-letter and 2-letter country code list. Since Kosovo has no standardized ISO 3 code, we use XKX and XK for Kosovo's policies. Similarly, we use SOL and XS for Somaliland. We use EUR and EU for Schengen policies.

For policies restricting access from countries with active COVID-19 cases that do not explicitly list countries that are restricted, we use the shorthand of COVIDCASES in the appropriate target field associated with partial bans.

3.4 RA Policy Search

In addition to coding the policies assigned by the RA Manager, RAs completed a weekly systematized self-search for new policies instituted by their assigned nations and territories. This self-search constituted the second and most important data-generating process for COBAP's database. The resources listed were assigned by an RA Manager and changed from week-to-week.

To complete the weekly review, the RA completed a Google search for travel restrictions related to each of their assigned countries. For a given country, the RA would first explore the Official Coronavirus response website for updates on COVID-19 related travel restrictions. If there was no designated Coronavirus site available, the RA would refer to the official governing body responsible for instituting and communicating travel and immigration policies. Once the RA had thoroughly scanned these government sources, s/he would scour the following, which are listed here in order of reliability:

[1] "[Although] Andorra is not part of the Schengen Area, border controls are carried out at its borders with the neighbouring Schengen states, France and Spain. However, Andorra coordinates its visa requirements with the Schengen Area and accepts Schengen visas (Source)." As such, the COBAP database records Schengen recommendations with Andorra included.

[2] Schengen policies introduced during the COVID-19 pandemic stated "Residents of Andorra, Monaco, San Marino and the Vatican should be considered as EU residents for the purpose of this recommendation." For this reason, any Schengen policy exception list count includes these countries.

Proxy Sources, in order of priority

1. Airline Industry (IATA)
2. International Insurance Agency (SOS)
3. Picture of policy text posted on an official social media venue of the host country (Twitter or Facebook). The RA must navigate to these pages from the host government's official website.

External Sources

4. Government website of an external country

Media sources

5. Media: major news outlet of host country
6. Media: major news outlet of an external country

3.5 Policy Assignments from the RA Manager

Each week, an RA manager identified new resources for border closures and allocated new policies to RAs based on the countries they were assigned. For example, each week s/he downloaded and restricted the international-level restrictions on movement from the ACAPS dataset (found here) to depict correct dates and more consistently categorize information on international immigration-related policies. These policies were assigned weekly to research assistants to interpret and enter, when applicable, into the COBAP database. Below is the process followed.

First, the RA manager downloaded the most recently updated ACAPS dataset.

The "Measure" category was filtered to display only: "Border closure", "complete border closure", "International flight suspension", and "visa restrictions", as these subcategories directly correlate to the project's conceptual framework.

To remove non-international policies, the RA manager filtered out from "ADMIN_LEVEL_NAME" any policy which is populated with content.

For purposes of restricting data to focus on international borders, the RA manager collapsed the columns: "region", "PCODE" and "category".

At this point, the policies were organized, first with ACAPS assigned IDs, by country; the RA Manager pasted policies to the RA Assignment Spreadsheet according to RA country assignments, keeping the columns POLICY ID, COUNTRY, ISO, ENTRY DATE, SOURCE 1, SOURCE 2, MEASURE). The columns COMMENTS, POLICY CODED, and CODERS COMMENTS were added to the sheet to keep track of the RA's decision making process for inclusion of each

policy, based on COBAP's pre-set definitions.

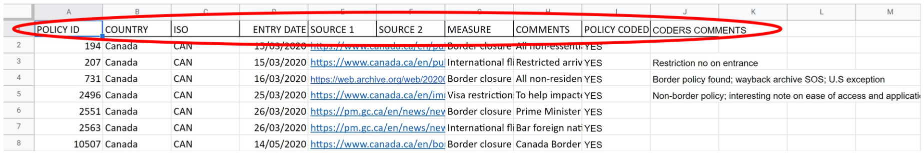

	POLICY ID	COUNTRY	ISO	ENTRY DATE	SOURCE 1	SOURCE 2	MEASURE	COMMENTS	POLICY CODED	CODERS COMMENTS
2	194	Canada	CAN	15/03/2020	https://www.canada.ca/en/pu		Border closure	All non-essenti	YES	
3	207	Canada	CAN	15/03/2020	https://www.canada.ca/en/pu		International fl	Restricted arriv	YES	Restriction no on entrance
4	731	Canada	CAN	16/03/2020	https://web.archive.org/web/2020(		Border closure	All non-residen	YES	Border policy found; wayback archive SOS; U.S exception
5	2496	Canada	CAN	25/03/2020	https://www.canada.ca/en/imi		Visa restriction	To help impacte	YES	Non-border policy; interesting note on ease of access and applicati
6	2551	Canada	CAN	26/03/2020	https://pm.gc.ca/en/news/nev		Border closure	Prime Minister	YES	
7	2563	Canada	CAN	26/03/2020	https://pm.gc.ca/en/news/nev		International fl	Bar foreign nati	YES	
8	10507	Canada	CAN	14/05/2020	https://www.canada.ca/en/bo		Border closure	Canada Border	YES	

Figure 1: Example Assignment sheet with RA Manager-added policies.

Once all policies were designated to the appropriate RAs, the RA Manager posted to the team Slack channel alerting RAs of new assignments.

3.6 Assignment Sheets for RAs

When an RA located an unassigned policy, s/he first recorded the policy in his/her assignment sheet, including information for each of the following columns: POLICY ID, COUNTRY, POLICY TYPE, ENTRY DATE, SOURCE 1, MEASURE.

POLICY ID: The RA generated a new policy ID with their first and last initial, and an integer counting up from 1. Ex: MS1, MS2, and MS3 are three distinct policy IDs. [After April 15, 2021 we introduced a standardized alphanumeric policy ID to correspond with ISO country codes.]

COUNTRY NAME: The RA referred to COBAP's country list (3.1) for correct spelling.

POLICY TYPE: The RA notes whether the new policy was a completely new policy and/or an "End Date" to a past policy.

SOURCE: The RA recorded the source text in which the policy is described. This sources were manually archived via the Wayback Machine.

MEASURE: The RA commented during their initial interpretation process of the measure. Is it a complete border closure? Partial closure? Does it appear targeted? Are individuals of specific nationalities restricted access or exempted from a ban? These observations will help to streamline the data entry process via Qualtrics.

RAs also made note of policy assignments added by the RA manager (see Figure 1), and assessed the policy type, measure, and source for each.

RAs began each coding session for a new policy by referring to the existing data, filtering on the country under review to ensure the policy has not already been coded, e.g by checking for the same start date, policy measures, and ID. If, during this process, an inaccuracy was identified in a previously recorded policy,

the RA fixed the error by re-entering the policy via Qualtrics and informed the main slack channel with the POLICY ID that s/he did so.

4 Qualtrics Surveys

For missing data, the policies were entered into the database using two Qualtrics surveys: survey 1, for entering new policies, and survey 2, for entering end dates or extensions of existing policies.

New entries were needed when countries (1) introduced a new measure, (2) ended existing measures, (3) extended existing measures, or (4) changed an existing measure into a new restriction. In this section, we explain the logic and uses of each survey (4.1 and 4.2) and the process for using both (4.3).

4.1 Qualtrics survey 1: adding new policies

Qualtrics survey 1 was used to enter new policies into the database. Survey 1 prompted the RA to answer a series of questions to categorize the policy by its scope. The RA completed survey 1 per policy, refreshing the link and starting the survey over for each policy record. The survey begins with a prompt to indicate (1) who you are (by selecting your email address from a drop-down menu); (2) a new policy ID; and (3) the country name for the policy being reviewed.

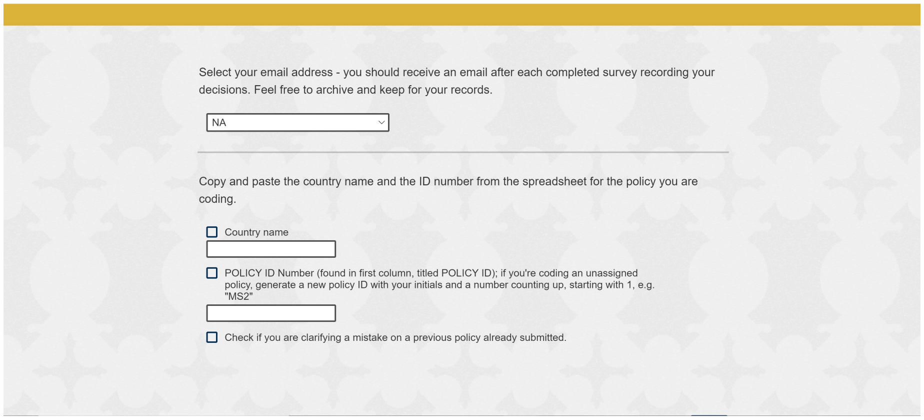

Figure 2: Qualtrics Survey 1

If the policy restricts entry over an international border, the RA indicated the source type and included a Wayback Machine-generated archive link.

Each policy was initially categorized as a Complete Closure or a Partial Closure, with 10 as the break-off number for countries which can be exceptions to a complete closure. If a policy bars 11 or more countries from entering, it was

recorded as a partial closure. Next, the policy's start and end dates (if known) were entered.

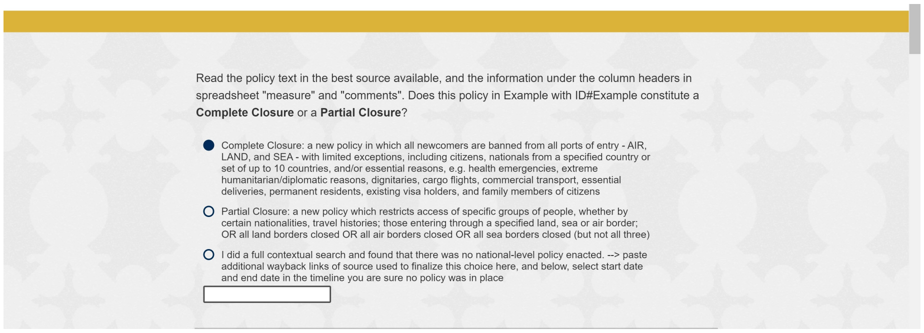

Figure 3: Entering Complete or Partial Closure via Qualtrics Survey 1.

After the decision between a complete and partial closure, there are two possible routes of continued questioning, depending upon whether the RA selects the complete or partial closure definition:

1. If the policy was a **Complete Closure**, the Qualtrics survey directed the RA to input any exceptions to the entry restrictions. These exceptions were divided into those made for citizen, workers, or specific countries. Note: there could be any combination of these exceptions.

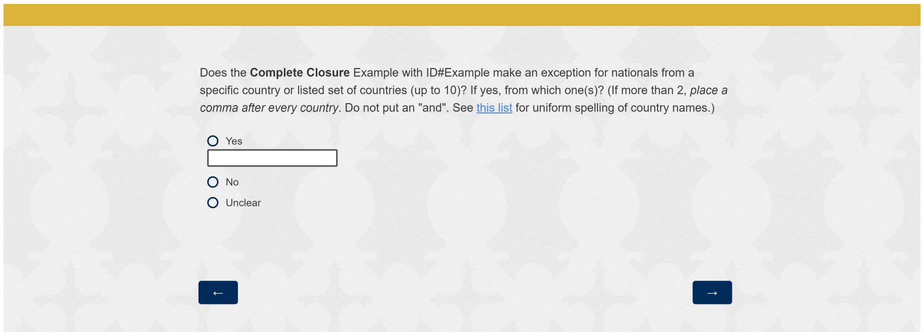

Figure 4: Example of exceptions entry via Qualtrics Survey 1.

2. If the policy was a Partial Closure, the Qualtrics survey directed the RA to further categorize the restriction as a border-based, status-based, or visa-based ban.

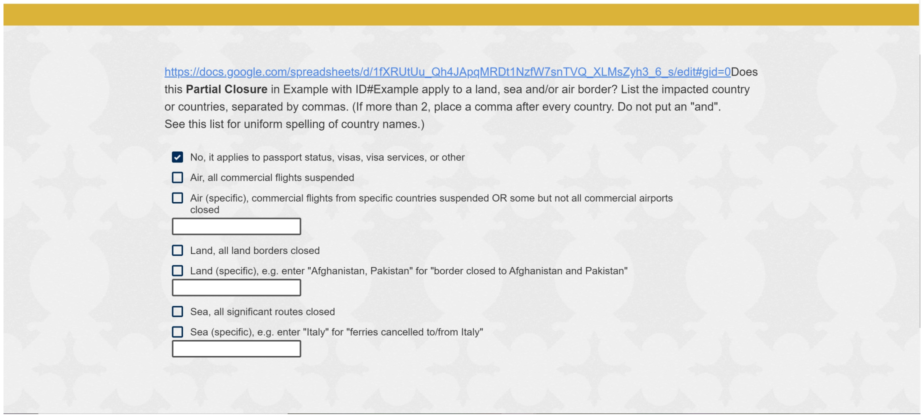

https://docs.google.com/spreadsheets/d/1fXRUtUu_Qh4JApqMRDt1NzfW7snTVQ_XLMsZyh3_6_s/edit#gid=0Does this **Partial Closure** in Example with ID#Example apply to a land, sea and/or air border? List the impacted country or countries, separated by commas. (If more than 2, place a comma after every country. Do not put an "and". See this list for uniform spelling of country names.)

- [x] No, it applies to passport status, visas, visa services, or other
- [ ] Air, all commercial flights suspended
- [ ] Air (specific), commercial flights from specific countries suspended OR some but not all commercial airports closed
- [ ] Land, all land borders closed
- [ ] Land (specific), e.g. enter "Afghanistan, Pakistan" for "border closed to Afghanistan and Pakistan"
- [ ] Sea, all significant routes closed
- [ ] Sea (specific), e.g. enter "Italy" for "ferries cancelled to/from Italy"

Figure 5: Example entry of partial border closure via Qualtics Survey 1.

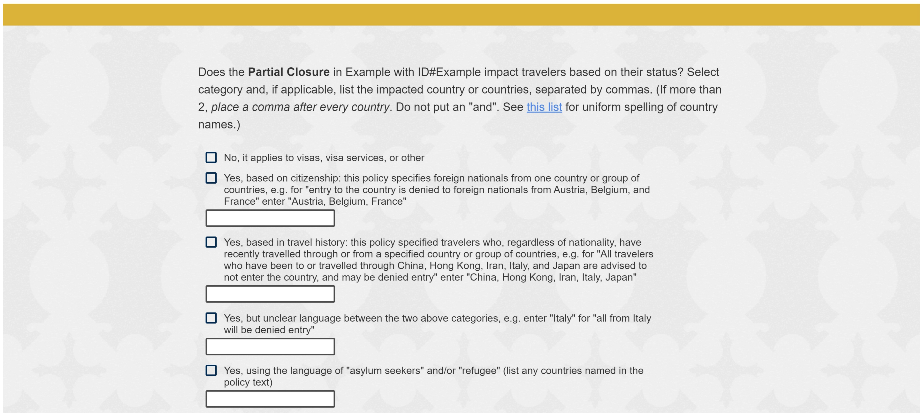

Does the **Partial Closure** in Example with ID#Example impact travelers based on their status? Select category and, if applicable, list the impacted country or countries, separated by commas. (If more than 2, *place a comma after every country*. Do not put an "and". See this list for uniform spelling of country names.)

- [ ] No, it applies to visas, visa services, or other
- [ ] Yes, based on citizenship: this policy specifies foreign nationals from one country or group of countries, e.g. for "entry to the country is denied to foreign nationals from Austria, Belgium, and France" enter "Austria, Belgium, France"
- [ ] Yes, based in travel history: this policy specified travelers who, regardless of nationality, have recently travelled through or from a specified country or group of countries, e.g. for "All travelers who have been to or travelled through China, Hong Kong, Iran, Italy, and Japan are advised to not enter the country, and may be denied entry" enter "China, Hong Kong, Iran, Italy, Japan"
- [ ] Yes, but unclear language between the two above categories, e.g. enter "Italy" for "all from Italy will be denied entry"
- [ ] Yes, using the language of "asylum seekers" and/or "refugee" (list any countries named in the policy text)

Figure 6: Example entry of Citizenship or Travel History ban via Qualtrics Survey 1.

4.2 Qualtrics Survey 2: adding end dates

If the RA discovered a policy which ended an already coded policy, it was entered into the database via Qualtrics Survey 2. The second Qualtrics survey first prompted the RA to input the country and ID of the policy ended by the new policy (see appendix 8.2 for full text). *If the new policy simply ended the previously entered policy*, i.e there were no new measures implemented by the new policy, *there was no need to enter the ID of the new policy*. That is, an end date for the previous policy was coded without entering a new policy into the database. *If the new policy ended the old policy* and *introduced new or changed measures*, the policy ID and archived source link of the superseding policy were entered.

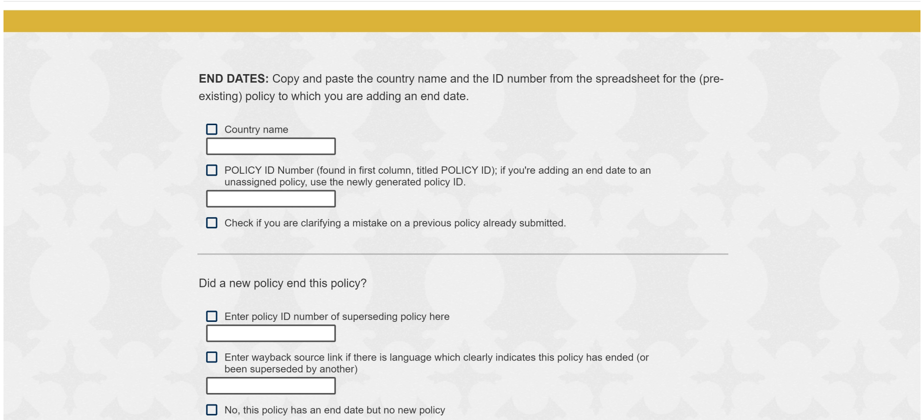
END DATES: Copy and paste the country name and the ID number from the spreadsheet for the (pre-existing) policy to which you are adding an end date.

- Country name
- POLICY ID Number (found in first column, titled POLICY ID); if you're adding an end date to an unassigned policy, use the newly generated policy ID.
- Check if you are clarifying a mistake on a previous policy already submitted.

Did a new policy end this policy?

- Enter policy ID number of superseding policy here
- Enter wayback source link if there is language which clearly indicates this policy has ended (or been superseded by another)
- No, this policy has an end date but no new policy

Figure 7: End date policy entry via Qualtrics Survey 2.

4.3 Using Both Surveys 1 and 2 to Record Policy Changes

Policies dropped into RAs' individual assignment sheets typically introduced new measures, ended or extended existing measures, or phased out different measures. It was the task of the RA to decide whether to include them in the COBAP database. The RA used Survey 1 if the policy was not yet recorded in the database and Survey 2 if s/he was updating a previous policy with an end date. In some cases, a given source constituted both an end date to a previous policy and a new policy.

For instance, Romania introduced (and continues to introduce at time of writing) a weekly revolving list of countries that are allowed in. We recorded each of these policy texts as an end date for the previous policy and as a new policy with the updated list of countries restricted.

Note: for partial closures (more than 10 countries banned), the database records the list of countries restricted not the ones allowed in.

5 RAs Frequently Asked Questions (FAQs)

1. *Do I code additional restrictions on entry, such as required quarantines or extra screening procedures targeted at certain nationalities?*

   No; we only record cross-border restrictions on entry.

2. *A country's population can transit through an otherwise closed country, but are not permitted to enter and stay. How do I code this?*

   Record this as a complete closure. Do not record the transit as an exception for the countries which can transit.

3. *All neighboring countries closed their borders with a country and effectively led to a border closure around the country. How do I code this?*

Only record the policy for a given country if the country's national government implemented the policy.

4. *How do I code multiple phase-out measures for one policy?*

   Recorded this as a new policy and an end date for the previous policy, provided some part of the restriction was still in place. For instance, if a country under a complete closure introduces an exception for another country, the RA records a new policy, adding the new country excepted to the list of country exceptions–entering via survey 2 the start date of the new policy as the end date of policy initially recorded. The next phase-out measure introduced by the same country was recorded in the same way.

5. *I found a better source for a policy I have already coded. How can I change this in the database?*

   If a better source is found with the official policy text (or description of the policy) of an already coded measure, re-code the policy via Qualtrics Survey 1, selecting "check if you are clarifying a mistake on a previous policy already submitted". Make sure to include the archived link to the better source when prompted.

6. *One of my assigned countries instituted the same policy multiple times throughout the year. Should I code the same policy multiple times?*

   Yes; enter the policy into the database, making note of each new start date.

7. *My policy source text does not include a list of countries from which travel is prohibited; instead, it includes a list of countries allowed entry. How do I code for this?*

   These are tricky. To code correctly, first, verify the number of country exceptions. Per our definitions, if the country allows entry for 10 or fewer countries, record as a complete_closure (and list the country exceptions. If the list contains more than 10 countries, code the policy as a partial_closure, and enter via the COBAP country list, the names of countries not allowed entry.

8. *One of my assigned countries introduced a policy prohibiting their citizens from travelling to another country. How do I code for this?*

   COBAP only records policies which restrict entry into the host country, so we do not need to record this information unless it prohibits entry from the target country.

9. *One of the policies I previously coded appears to have been coded incorrectly. How do I fix this error?*

   Re-code the policy using the appropriate Qualtrics survey. If the mistake was an end date, re-code the end date using Qualtrics Survey 2. If the mistake is related to the policy measures, re-code the policy using Qualtrics Survey 1. In either case, make sure to select the box "Check if clarifying a mistake on a previous policy already submitted."

10. *I just discovered an end date for a policy I already coded. Do I need to re-code the policy?*

    No; an end date can be added to a previously recorded policy via Qualtrics Survey 2. If a new policy phases out the previous policy, or introduces more strict measures, be sure to include its ID under "Enter policy ID number of superseding policy"; if there was no new policy implemented, leave this section empty.

11. *How do I code a policy extension if no substantive change has been made to the policy?*

    If no change has been made to the policy, re-code the policy end date using Qualtrics Survey 2, check the box "Check if clarifying a mistake on a previous policy already submitted", and code the new end date where prompted.

12. *I have three partial closures that individually close land, air, and sea borders. What source should I use to code this as a complete closure?*

    Record these separately with the best possible source for each one.

13. *How do I code a new phase-out measure for a complete closure if it opens one or two (but not all) border types?*

    If the phase-out measure for a complete closure opens one or more border types (land, air, or sea), the phase-out policy should be coded as a partial closure, closing the border types not re-opened by the phase-out. Make sure to enter the start date of the phase- out policy as the end date of the complete closure via Qualtrics Survey 2, entering the superseding policy ID as you code.

14. *How do I code a new phase-out measure for a complete closure if it permits entry of more than 10 countries?*

    If the phase-out measure for a complete closure makes an exception for more than 10 countries, the phase-out policy should be coded as a partial closure, restricting entry from all countries not allowed entry. Make sure to enter the start date of the phase-out policy as the end date of the complete closure via Qualtrics Survey 2, entering the superseding policy ID as you code. Then, record the superseding policy ID with the list of countries not allowed (not the ones allowed in). The fastest way to do this if a small list is to copy and paste from the country list above (3.1) and remove the countries named as excepted from the ban.

15. *How do I record data for a country whose territory is disputed, for instance, Western Sahara – between the Sahrawi Arab Democratic Republic and Morocco?*

    For the Western Sahara country code, record data that corresponds with policies introduced by the Sahrawi Arab Democratic Republic's Polisario Front; for Morocco's country code, record data introduced by Morocco.

16. *How do I record targeted countries for EU policies that allow only EU and Schengen Area nationals to travel, do I have to list all other countries?*

    For policies that explicitly allow travel from European nations only, enter the code NONEU to represent all countries except those considered within the Schengen Area (see Section 3.2), this will be automatically replaced during processing to include all other countries.

6 Contributor Expectations

The COBAP team recognizes the extraordinary effort and time commitment that RAs volunteered to the collection and maintenance of the COBAP database. Without these volunteers, the COBAP public resource would not be possible. As a part of their commitment to contribute to the dataset, RAs held themselves to the following expectations:

1. Completed necessary training and agreement through Google Form 2.1 to update their assigned countries each week and communicate with the project at least once per week, responding to time-sensitive questions within 24 hours M-F;
2. Entered was done so with the goal to reflect the true policy timeline (with mistakes fixed in a timely manner);
3. Reviewed her assigned and other RAs' policies for inconsistencies, errors, and accuracy;
4. Communicated questions and concerns regarding confusing or complex policies with the COBAP team via Slack (for quick questions) or email (for analysis or outreach-related questions);
5. Submitted, via Country Updates (Google Form 2.3), a weekly record of policies entered for each assigned country, even if they found no new policies were added or changed for a given country, and;
6. Communicated with the COBAP team when unable to meet weekly data collection responsibilities.

To acknowledge the hard work and dedication of the COBAP RAs, each had the opportunity to be listed as a coauthor of the dataset. The following details the necessary requirements for coauthorship:

1. All contributing RA authors completed at least 20 hours of data collection and validation;
2. All contributing RA authors completed data collection or validation for at least 5 countries, covering the complete timeline of 2020;
3. All contributing authors read and approved of each manuscript submission, suggesting edits as they saw fit (using Google Form 2.4 for first submission); and

4. All contributing authors verified their confidence in the data for each assigned country, signing off on the final manuscript (using Google Form 2.6).

7 The Names behind the Data

Liz Beling was responsible for collecting and/or verifying the policies of the following countries for the 2020 timeline: Antigua and Barbuda, Bahamas, Bahrain, Barbados, Cook Islands, Cyprus, Dominican Republic, East Timor, Northern Mariana Islands, Puerto Rico, Guam, Svalbard and Jan Mayen, U.S. Virgin Islands, Antarctica, Andorra, Aland Islands, Bonaire, Bouvet Islands, Federated States of Micronesia, Fiji, Grenada, Jamaica, Kiribati, Maldives, Guernsey, Gibraltar, Anguilla, Bermuda, British Indian Territory, Cayman Islands, Falkland Islands, Pitcairn (Islands), Isle of Man, Jersey, Montserrat; Saint Helena, Ascension and Tristan da Cunha, Turks and Caicos Islands, Virgin Islands (British), Saint Vincent and the Grenadines, United States Minor Outlying Islands, Montenegro, Seychelles, Solomon Islands, Tonga, Trinidad and Tobago, Tuvalu, Vanuatu, Aruba, Curaçao, Sint Maarten (Dutch part), Faroe Islands, Martinique, and Greenland.

Cayleigh Jackson was responsible for: China, Hong Kong, Macau, Malaysia, Taiwan, Singapore, and Tibet.

Mark A. Weiss was responsible for: Saudi Arabia, Russia, Iran, Jordan, UAE, Oman, Qatar, Syria, Turkey, North Korea, Venezuela, Yemen, Central African Republic, Eritrea, Cuba, Libya and India.

Noah Rusk Taylor was responsible for: Togo, Senegal, Saint Pierre and Miquelon, Saint Martin (French part), Saint Barthélemy, Rwanda, Réunion, Niger, Mayotte, Mali, Guinea, Gabon, Equatorial Guinea, Djibouti, Democratic Republic of the Congo, Congo, Comoros, Chad, Burundi, Burkina Faso, Côte d'Ivoire, French Guiana, and French Polynesia.

Cora Hirst was responsible for: United Kingdom, Dominica, Haiti, Saint Lucia, France, French Polynesia, New Caledonia, Wallis and Futuna, Saint Pierre and Miquelon, Saint Martin (French part), Viet nam, Myanmar (Burma), Thailand, Lao, Cambodia, Indonesia, Sri Lanka, Bangladesh, Bhutan, Mongolia, Madagascar, the Caribbean Netherlands and Israel.

Layth Mattar was responsible for: Iraq, Palestine, Tajikistan, Georgia, Lebanon, Sierra Leone, South Africa, Namibia, Zimbabwe, Tanzania, Uganda, South Sudan, Eswatini, Libya, Western Sahara, Liberia, Somaliland, Morocco, Western Sahara, Botswana, Lesotho, Brunei Darussalam, and Pakistan.

Nikolas Lazar was responsible for: Sudan, Benin, Kenya, Somalia, Ghana, Nigeria, Cameroon, Egypt, Gambia, Zambia, Malawi, American Samoa and Co-

cos/Keeling, Australia, Christmas Island, Norfolk Island, Nauru, Japan, Greece, Poland, and Guadaloupe.

William Yu was responsible for: Malta, Marshall Islands, Mauritius, Niue, Palau, Papua New Guinea, the Philippines, Saint Kitts and Nevis, Samoa, and Tokelau.

Sarah Naseer was responsible for: Sweden, Slovakia, Latvia, Angola, Cape Verde, Guinea-Bissau, Mozambique, Sao Tome and Principe, Martinique, Wallis and Futuna, Panama, and Belize.

Erin Straight was responsible for: Nicaragua, Guatemala, El Salvador, Spain, Colombia, Brazil, Paraguay, Bolivia, Ecuador, Mexico, Portugal, United States, Moldova, Ukraine, Kazakhstan, Kyrgyzstan, Afghanistan, Bosnia and Herzegovina, Bulgaria, Belarus, Argentina, and Kuwait.

Jonathan Falcone was responsible for: Vatican City, Iceland, Uzbekistan, Turkmenistan, South Korea, Croatia, Tunisia, Algeria, and Norway.

Mary Louise Mitsdarffer was responsible for: Italy, Uruguay, Peru, Chile, and Romania.

Lukas Feddern was responsible for: EU/Schengen, Azerbaijan, Germany, Austria, Liechtenstein, Switzerland, Luxembourg, the Netherlands, Belgium, Armenia, Nepal, Slovenia, Lithuania, Finland, Czechia, Denmark, Estonia, Hungary, San Marino, Monaco, and Ireland.

Sonila Hasaj was responsible for Kosovo, Serbia, Albania, Mauritania, North Macedonia, and Mauritius.

Aadya Bhashakran, Rachel Musetti, Amalia Gradie, Bryn Walker, Hannah Rossi, Hannah Risman, Michael Shiraef, Ellen Shiraef, Aman Bedi, Camilla Kline and Suzanne Martin collected data for at least five countries during a portion of the 2020 timeline.

This document was drafted and curated by Cora Hirst.

8 Appendix

8.1 Full Text of Qualtrics Survey 1

Coding_Country-Level_Immigration_Policy_Responses_to_Covid-19

Start of Block: Default Question Block

Q29 Select your email address - you should receive an email after each completed survey recording your decisions. Feel free to archive and keep for your records.

▼ mshiraef@nd.edu (25) ... NA (24)

Q1 Copy and paste the country name and the ID number from the spreadsheet for the policy you are coding.

☐ Country name (4) ____

☐ POLICY ID Number (found in first column, titled POLICY ID); if you're coding an unassigned policy, generate a new policy ID with your initials and a number counting up, starting with 1, e.g. "MS2" (5) ____

☐ Check if you are clarifying a mistake on a previous policy already submitted. (7)

Q24 At first glance, is this an international-level policy which relates (in any way) to a restriction on people outside the country attempting to travel inside of the country? For assigned policies, read the information under the column headers in spreadsheet "measure" and "comments" in ${Q1/ChoiceTextEntryValue/4} with ID#${Q1/ChoiceTextEntryValue/5}. (For non-assigned policies, assess the policy text in a reliable source.)

- ◯ Yes/Unsure, e.g. "Saudi Arabia bans all foreigners and citizens from entering" (1)
- ◯ No, it is only outgoing, e.g. "Saudi Arabia bans Saudi citizens from traveling traveling to Iran, China, and Belgium" (2)
- ◯ No, it is only an internal restriction, e.g. Saudi Arabia restricts residents from x region from traveling to y region". (7)
- ◯ No, it is none of the above. (8)

Display This Question:

If At first glance, is this an international-level policy which relates (in any way) to a restrictio... = Yes/Unsure, e.g. "Saudi Arabia bans all foreigners and citizens from entering"

*

Q2

For ${Q1/ChoiceTextEntryValue/4} with ID#${Q1/ChoiceTextEntryValue/5}, click on the link under SOURCE 1 (and under SOURCE 2 if there is one) and record the source-type. If two sources are included, select both source-types below.

- ☐ 1. Government: website of the host country ending in .gov (in a language I can read) or an official two letter domain abbreviation, e.g. "embassy.gr" [IDEAL SOURCE] (1)
- ☐ 2. Airline Industry: IATA [PROXY SOURCE] (12)
- ☐ 3. International Insurance Industry: SOS [PROXY SOURCE] (2)
- ☐ 4. Government: verified social media post [PROXY SOURCE] (11)
- ☐ 5. Government website of an external country ending in .gov [EXTERNAL SOURCE] (9)
- ☐ 6. Media: major news outlet of host country [INTERNAL MEDIA SOURCE] (6)
- ☐ 7. Media: major news outlet of external country [EXTERNAL MEDIA SOURCE] (7)
- ☐ 8. Other organizations / I'm unsure / no link / policy text not found and/or link doesn't work [NON-STATE ORGANIZATION] (15)
- ☐ 9. The source is in a language other than English. (16)

Display This Question:

If At first glance, is this an international-level policy which relates (in any way) to a restrictio... = Yes/Unsure, e.g. "Saudi Arabia bans all foreigners and citizens from entering"

And For ${q://QID13/ChoiceTextEntryValue/4} with ID#${q://QID13/ChoiceTextEntryValue/5}, click on the... = 9. The source is in a language other than English.

Q28 If you're unable to read the language of the policy text, but think it may be a reputable source, try to find a snapshot of the link on wayback machine by copying and pasting the link in the search browser, on a date on/before the listed ENTRY DATE. (If there are no snapshots on/before, you can use any snapshot which shows the policy text or snap the page yourself). You can try to search for the policy text via a Google Translate function in your browser (or by copying and pasting the relevant text into Google Translate). If the translation is not clear, you can check with one of our project's linguistic experts (Cayleigh/Dallas for Chinese, Cora/Noah for French, Aadya/Yeshwini/Boovhita for Hindi, Lillith for German, Mary for Greek/Thai, Yeshwini for Japanese, Yeshwini for Kannada, Thuy for Vietnamese and Yeshwini for Tamil/Boovhita.)

○ Found it (1) ____

○ wayback machine did not record a snapshot, or snapshot is empty in content, or the source does not look reliable (2)

Display This Question:

If At first glance, is this an international-level policy which relates (in any way) to a restrictio... = Yes/Unsure, e.g. "Saudi Arabia bans all foreigners and citizens from entering"

And For ${q://QID13/ChoiceTextEntryValue/4} with ID#${q://QID13/ChoiceTextEntryValue/5}, click on the... = 1. Government: website of the host country ending in .gov (in a language I can read) or an official two letter domain abbreviation, e.g. "embassy.gr" [IDEAL SOURCE]

Or For ${q://QID13/ChoiceTextEntryValue/4} with ID#${q://QID13/ChoiceTextEntryValue/5}, click on the... = 2. Airline Industry: IATA [PROXY SOURCE]

Or For ${q://QID13/ChoiceTextEntryValue/4} with ID#${q://QID13/ChoiceTextEntryValue/5}, click on the... = 3. International Insurance Industry: SOS [PROXY SOURCE]

Or For ${q://QID13/ChoiceTextEntryValue/4} with ID#${q://QID13/ChoiceTextEntryValue/5}, click on the... = 4. Government: verified social media post [PROXY SOURCE]

Q27 Locate a link to the snapshot on wayback machine of the source you're recording on/before the date the policy was recorded (date found under column header "ENTRY DATE"). If there

is none, snap one yourself
Keep in mind the dates in your assignment spreadsheet automated into a DD/MM/YYYY format (whereas the policy source you've found might be in either MM/DD/YYYY format or DD/MM/YYYY format).

- [ ] wayback snapshot link to IATA (1)

____

- [ ] wayback snapshot link to SOS (2)

____

- [ ] wayback snapshot link of actual policy and/or description of it at a .gov website of the host country (3)

____

- [ ] wayback snapshot link to verified social media post (4)

____

Display This Question:

If For ${q://QID13/ChoiceTextEntryValue/4} with ID#${q://QID13/ChoiceTextEntryValue/5}, click on the... = 5. Government website of an external country ending in .gov [EXTERNAL SOURCE]

Or For ${q://QID13/ChoiceTextEntryValue/4} with ID#${q://QID13/ChoiceTextEntryValue/5}, click on the... = 6. Media: major news outlet of host country [INTERNAL MEDIA SOURCE]

Or For ${q://QID13/ChoiceTextEntryValue/4} with ID#${q://QID13/ChoiceTextEntryValue/5}, click on the... = 7. Media: major news outlet of external country [EXTERNAL MEDIA SOURCE]

Or For ${q://QID13/ChoiceTextEntryValue/4} with ID#${q://QID13/ChoiceTextEntryValue/5}, click on the... = 6. Media: major news outlet of host country [INTERNAL MEDIA SOURCE]

Or For ${q://QID13/ChoiceTextEntryValue/4} with ID#${q://QID13/ChoiceTextEntryValue/5}, click on the... = 8. Other organizations / I'm unsure / no link / policy text not found and/or link doesn't work [NON-STATE ORGANIZATION]

Or For ${q://QID13/ChoiceTextEntryValue/4} with ID#${q://QID13/ChoiceTextEntryValue/5}, click on the... = 9. The source is in a language other than English.

And If

For ${q://QID13/ChoiceTextEntryValue/4} with ID#${q://QID13/ChoiceTextEntryValue/5}, click on the... != 1. Government: website of the host country ending in .gov (in a language I can read) or an official two letter domain abbreviation, e.g. "embassy.gr" [IDEAL SOURCE]

And If

For ${q://QID13/ChoiceTextEntryValue/4} with ID#${q://QID13/ChoiceTextEntryValue/5}, click on the... != 4. Government: verified social media post [PROXY SOURCE]

And If

For ${q://QID13/ChoiceTextEntryValue/4} with ID#${q://QID13/ChoiceTextEntryValue/5}, click on the... != 3. International Insurance Industry: SOS [PROXY SOURCE]

And For ${q://QID13/ChoiceTextEntryValue/4} with ID#${q://QID13/ChoiceTextEntryValue/5}, click on the... != 3. International Insurance Industry: SOS [PROXY SOURCE]

And If

For ${q://QID13/ChoiceTextEntryValue/4} with ID#${q://QID13/ChoiceTextEntryValue/5}, click on the... != 2. Airline Industry: IATA [PROXY SOURCE]

And If

At first glance, is this an international-level policy which relates (in any way) to a restrictio... = Yes/Unsure, e.g. "Saudi Arabia bans all foreigners and citizens from entering"

Q20

For policy in ${Q1/ChoiceTextEntryValue/4} with ID#${Q1/ChoiceTextEntryValue/5}, are you able to find a second source which falls into one of the below categories? If so, copy and paste the link with the appropriate source type (link not necessary for IATA/SOS). Search in the following order:

1. Search briefly (not extensively) via Google for the actual policy text found at a government

website of the host country (ending in **.gov** or official two letter domain abbreviation, e.g. "embassy.**gr**"). Start the search with the COVID-19travel restrictions page on Wikipedia, which has a relevant .gov site for many countries. Try searching the Wiki page via Wayback as well (there are four snapshots during the month of May).
2. Next, [if the policy was implemented on/before Aug 5] search for air travel related policies and new visa policies, search IATA (via wayback); and [if the policy was implemented on/before May 27] for land and sea border policies, search International SOS (via wayback). Be sure to only capture Wayback links on/before these listed dates.
3. Locate (through google searches and media coverage) a social media post announcing the policy, from a verified account of host country's department of government.
5. Search a government website of an external country (ending in .gov), e.g. US State department. This list from the Overseas Advisory Council (OSAC) often has links to the policy from the host country website, and has an archive of recent "reports" which have helpful policy descriptions.
6. Use a Google search to locate the policy in a major news outlet of the host country
7. Use a Google search to locate the policy in a major news outlet of an external country, e.g. restrictions lists provided by Al Jazeera (via wayback) or New York Times (via wayback), or other coverage.

☐ Paste wayback link to IATA (1)

____

☐ Paste wayback link to SOS (2)

____

☐ Paste wayback snapshot link of actual policy and/or description of it at a .gov website of the host country (3)

____

☐ Paste wayback link to verified social media post here (4)

____

☐ Paste wayback link to website of external country here (5)

____

☐ Paste wayback link to to major news outlet of the host country here (6)

____

☐ Paste wayback link to a major news outlet of an external country here (14)
____

☐ I found a reptuable source verifying the policy text, but wayback hasn't snapshotted it. Snap and paste here: (16)
____

☐ Unable to find better source (if fits within our framework and you think the policy was in place, paste the source that helped you make your decision.) (15)
____

Display This Question:

If At first glance, is this an international-level policy which relates (in any way) to a restrictio... = Yes/Unsure, e.g. "Saudi Arabia bans all foreigners and citizens from entering"

Q5 Read the policy text in the best source available, and the information under the column headers in spreadsheet "measure" and "comments". Does this policy in ${Q1/ChoiceTextEntryValue/4} with ID#${Q1/ChoiceTextEntryValue/5} constitute a **Complete Closure** or a **Partial Closure**?

○ Complete Closure: a new policy in which all newcomers are banned from all ports of entry - AIR, LAND, and SEA - with limited exceptions, including citizens, nationals from a specified country or set of up to 10 countries, and/or essential reasons, e.g. health emergencies, extreme humanitarian/diplomatic reasons, dignitaries, cargo flights, commercial transport, essential deliveries, permanent residents, existing visa holders, and family members of citizens (1)

○ Partial Closure: a new policy which restricts access of specific groups of people, whether by certain nationalities, travel histories; those entering through a specified land, sea or air border; OR all land borders closed OR all air borders closed OR all sea borders closed (but not all three) (2)

○ I did a full contextual search and found that there was no national-level policy enacted. --> paste additional wayback links of source used to finalize this choice here, and below, select start date and end date in the timeline you are sure no policy was in place (3)
____

Display This Question:

If At first glance, is this an international-level policy which relates (in any way) to a restrictio... = Yes/Unsure, e.g. "Saudi Arabia bans all foreigners and citizens from entering"

Q3 **In which month was the policy first implemented?**
Locate this date within the policy text of your most reputable source
If no date is listed, check other source for a listed start date.
If no date is listed in other source, list the date on which the policy text was published of the most reputable source.
If no publication date is found in the most reputable source, record the publication date of the other source.
Keep in mind most policies around the world will be listed in a DD/**MM**/YYYY format, and you may have to combine information from the publication date of a source and the policy description, e.g. "Starting on Monday, no foreigners will be allowed."

▼ Jan (01) (1) ... Dec (12) (24)

Display This Question:

If At first glance, is this an international-level policy which relates (in any way) to a restrictio... = Yes/Unsure, e.g. "Saudi Arabia bans all foreigners and citizens from entering"

Q21 Which calendar day (1-31) was the policy first implemented? Keep in mind most policies around the world will be listed in a **DD**/MM/YYYY format. If you're not sure and need a second review, put "32"

○ Enter number (1-31) here (1) ____

Display This Question:

If At first glance, is this an international-level policy which relates (in any way) to a restrictio... = Yes/Unsure, e.g. "Saudi Arabia bans all foreigners and citizens from entering"

Q32 Which year was this policy first implemented? Keep in mind most policies around the world will be listed in a DD/MM/YYYY format.

○ 2020 (1)

○ 2021 (2)

Display This Question:

If At first glance, is this an international-level policy which relates (in any way) to a restrictio... = Yes/Unsure, e.g. "Saudi Arabia bans all foreigners and citizens from entering"

Q4 In which month did this policy **end** (or is set to end in the future)?
Locate this date within the policy text of your second source. If no date is listed, select "no end date found". Keep in mind most policies around the world will be listed in a DD/MM/YYYY format.

▼ Jan (01) (5) ... No end date found (16)

Display This Question:

If At first glance, is this an international-level policy which relates (in any way) to a restrictio... = Yes/Unsure, e.g. "Saudi Arabia bans all foreigners and citizens from entering"

Q22 Which calendar day (1-31) did this policy end (or is set to end in the future)? Keep in mind most policies around the world will be listed in a **DD**/MM/YYYY format.

○ Enter number 1-31 here (1) ____

○ No end date found (2)

Display This Question:

If At first glance, is this an international-level policy which relates (in any way) to a restrictio... = Yes/Unsure, e.g. "Saudi Arabia bans all foreigners and citizens from entering"

Q31 Which year didthis policy end (or is set to end in the future)? Keep in mind most policies around the world will be listed in a DD/MM/**YYYY** format.

○ 2020 (1)

○ 2021 (2)

Display This Question:

If Read the policy text in the best source available, and the information under the column headers i... = Complete Closure: a new policy in which all newcomers are banned from all ports of entry - AIR, LAND, and SEA - with limited exceptions, including citizens, nationals from a specified country or set of up to 10 countries, and/or essential reasons, e.g. health emergencies, extreme humanitarian/diplomatic reasons, dignitaries, cargo flights, commercial transport, essential deliveries, permanent residents, existing visa holders, and family members of citizens

And At first glance, is this an international-level policy which relates (in any way) to a restrictio... = Yes/Unsure, e.g. "Saudi Arabia bans all foreigners and citizens from entering"

Q6 Does this **Complete Closure** in ${Q1/ChoiceTextEntryValue/4} with ID#${Q1/ChoiceTextEntryValue/5} make a citizen-related exception (including citizens, permanent residents, and/or family members of citizens and permanent residents)? List separated by commas.

○ Yes (1) ____

○ No (2)

Display This Question:

If Read the policy text in the best source available, and the information under the column headers i... = Partial Closure: a new policy which restricts access of specific groups of people, whether by certain nationalities, travel histories; those entering through a specified land, sea or air border; OR all land borders closed OR all air borders closed OR all sea borders closed (but not all three)

And At first glance, is this an international-level policy which relates (in any way) to a restrictio... = Yes/Unsure, e.g. "Saudi Arabia bans all foreigners and citizens from entering"

Q10 Does this **Partial Closure** in ${Q1/ChoiceTextEntryValue/4} with ID#${Q1/ChoiceTextEntryValue/5} apply to a land, sea and/or air border? List the impacted country or countries, separated by commas. See this list for uniform spelling of country names.)

- ☐ ⊗No, it applies to passport status, visas, visa services, or other (5)
- ☐ Air, all commercial flights suspended (1)
- ☐ Air (specific), commercial flights from specific countries suspended OR some but not all commercial airports closed, e.g. enter "Indonesia" for "all access both inbound and outbound from airports in Papua" [specify Papua in the upcoming notes question] (6) ____
- ☐ Land, all land borders closed (7)
- ☐ Land (specific), e.g. enter "Afghanistan, Pakistan" for "border closed to Afghanistan and Pakistan" (3) ____
- ☐ Sea, all significant routes closed (4)
- ☐ Sea (specific), e.g. enter "Italy" for "ferries cancelled to/from Italy" (8) ____

Display This Question:

If Read the policy text in the best source available, and the information under the column headers i... = Partial Closure: a new policy which restricts access of specific groups of people, whether by certain

nationalities, travel histories; those entering through a specified land, sea or air border; OR all land borders closed OR all air borders closed OR all sea borders closed (but not all three)

And Does this Partial Closure in ${q://QID13/ChoiceTextEntryValue/4} with ID#... = No, it applies to passport status, visas, visa services, or other

And At first glance, is this an international-level policy which relates (in any way) to a restrictio... = Yes/Unsure, e.g. "Saudi Arabia bans all foreigners and citizens from entering"

Q7 Does the Partial Closure in ${Q1/ChoiceTextEntryValue/4} with ID#${Q1/ChoiceTextEntryValue/5} impact travelers based on their status? Select category and, if applicable, list the impacted country or countries, separated by commas. See this list for uniform spelling of country names.)

- ☐ ⊗ No, it applies to visas, visa services, or other (3)

- ☐ Yes, based on citizenship: this policy specifies foreign nationals from one country or group of countries, e.g. for "entry to the country is denied to foreign nationals from Austria, Belgium, and France" enter "Austria, Belgium, France" (1)
____

- ☐ Yes, based in travel history: this policy specified travelers who, regardless of nationality, have recently travelled through or from a specified country or group of countries, e.g. for "All travelers who have been to or travelled through China, Hong Kong, Iran, Italy, and Japan are advised to not enter the country, and may be denied entry" enter "China, Hong Kong, Iran, Italy, Japan" (2)
____

- ☐ ⊗ Yes, but unclear language between the two above categories, e.g. enter "Italy" for "all from Italy will be denied entry" (17)
____

- ☐ Yes, using the language of "asylum seekers" and/or "refugee" (list any countries named in the policy text) (18)
____

Display This Question:

If Read the policy text in the best source available, and the information under the column headers i... = Partial Closure: a new policy which restricts access of specific groups of people, whether by certain

nationalities, travel histories; those entering through a specified land, sea or air border; OR all land borders closed OR all air borders closed OR all sea borders closed (but not all three)

And Does this Partial Closure in ${q://QID13/ChoiceTextEntryValue/4} with ID#... = No, it applies to passport status, visas, visa services, or other

And Does the Partial Closure in ${q://QID13/ChoiceTextEntryValue/4} with ID#... = No, it applies to visas, visa services, or other

And At first glance, is this an international-level policy which relates (in any way) to a restrictio... = Yes/Unsure, e.g. "Saudi Arabia bans all foreigners and citizens from entering"

Q17 Does the Partial Closure in ${Q1/ChoiceTextEntryValue/4} with ID#${Q1/ChoiceTextEntryValue/5} impact new visa seekers?

- ○ No (1)
- ○ Yes, all new visa seekers, e.g. "all visa services are temporarily suspended" or "visa on arrival suspended" (2)
- ○ Yes, specific visa seekers, e.g. enter "China" for "visas suspended to foreign nationals from China" (3) ____

Display This Question:

If Read the policy text in the best source available, and the information under the column headers i... = Complete Closure: a new policy in which all newcomers are banned from all ports of entry - AIR, LAND, and SEA - with limited exceptions, including citizens, nationals from a specified country or set of up to 10 countries, and/or essential reasons, e.g. health emergencies, extreme humanitarian/diplomatic reasons, dignitaries, cargo flights, commercial transport, essential deliveries, permanent residents, existing visa holders, and family members of citizens

Q8 Does the **Complete Closure** in ${Q1/ChoiceTextEntryValue/4} with ID#${Q1/ChoiceTextEntryValue/5} make an exception for specific work permit status holders?

- ○ Yes (1)
- ○ No (2)
- ○ Unclear (3)

Display This Question:

If Read the policy text in the best source available, and the information under the column headers i... = Complete Closure: a new policy in which all newcomers are banned from all ports of entry - AIR, LAND, and SEA - with limited exceptions, including citizens, nationals from a specified country or set of up to 10 countries, and/or essential reasons, e.g. health emergencies, extreme humanitarian/diplomatic reasons, dignitaries, cargo flights, commercial transport, essential deliveries, permanent residents, existing visa holders, and family members of citizens

And At first glance, is this an international-level policy which relates (in any way) to a restrictio... = Yes/Unsure, e.g. "Saudi Arabia bans all foreigners and citizens from entering"

Q9 Does the **Complete Closure** ${Q1/ChoiceTextEntryValue/4} with ID#${Q1/ChoiceTextEntryValue/5} make an exception for nationals from a specific country or listed set of countries (up to 10)? If yes, from which one(s)? See this list for uniform spelling of country names.)

- ○ Yes (1) ____
- ○ No (2)
- ○ Unclear (3)

Q33 Notes or extra sources

- ○ Anything else we should know about this entry? (please keep info concise and clear) (1) ____
- ○ Any other sources you used for this decision? (please include waybacked link) (2) ____

End of Block: Default Question Block

8.2 Full Text of Qualtrics Survey 2

END DATES

Start of Block: Default Question Block

Q1 **END DATES:** Copy and paste the country name and the ID number from the spreadsheet for the (pre-existing) policy to which you are adding an end date.

- ☐ Country name (1) ____
- ☐ POLICY ID Number (found in first column, titled POLICY ID); if you're adding an end date to an unassigned policy, use the newly generated policy ID. (2) ____
- ☐ Check if you are clarifying a mistake on a previous policy already submitted. (3)

Q2 Did a new policy end this policy?

- ☐ Enter policy ID number of superseding policy here (1) ____
- ☐ Enter wayback source link if there is language which clearly indicates this policy has ended (or been superseded by another) (2) ____
- ☐ No, this policy has an end date but no new policy (3)

Q3 **What month did this policy end** (or is set to end in the future)**?**
If this policy was superseded by another, use the **start date** of the new policy as the end date below.
Keep in mind most policies around the world will be listed in a DD/MM/YYYY format.

▼ Jan (1) ... Click to write Choice 13 (13)

Q4 **Which calendar day (1-31) did this policy end (or is set to end in the future)?**
If this policy was superseded by another, use the same calendar day of the new policy as the end date below.
Keep in mind most policies around the world will be listed in a DD/MM/YYYY format.
[If you think this date decision needs to be reviewed, enter "32" and provide the source link which is confusing you.]

- Enter number 1-31 here (1) ____
- Enter source link for your date decision. (2) ____
- Enter any notes or clarifications which would explain to a user of the data why you recorded the date you did. (3)

Q5 Which year did this policy end?

- ○ 2020 (1)
- ○ 2021 (3)

End of Block: Default Question Block
